# Mineralocorticoid receptor promotes cardiac macrophage inflammaging

**DOI:** 10.1007/s00395-024-01032-6

**Published:** 2024-02-08

**Authors:** Daniela Fraccarollo, Robert Geffers, Paolo Galuppo, Johann Bauersachs

**Affiliations:** 1 Department of Cardiology and Angiology, Hannover Medical School, Carl-Neuberg-Str.1 30625, Hannover, Germany; 2 Research Group Genome Analytics, Helmholtz Centre for Infection Research, Brunswick, Germany

**Keywords:** Macrophage, Mineralocorticoid receptor, Aging, Inflammation, Fibrosis

## Abstract

Inflammaging, a pro-inflammatory status that characterizes aging and primarily involving macrophages, is a master driver of age-related diseases. Mineralocorticoid receptor (MR) activation in macrophages critically regulates inflammatory and fibrotic processes. However, macrophage-specific mechanisms and the role of the macrophage MR for the regulation of inflammation and fibrotic remodeling in the aging heart have not yet been elucidated. Transcriptome profiling of cardiac macrophages from male/female young (4 months-old), middle (12 months-old) and old (18 and 24 months-old) mice revealed that myeloid cell-restricted MR deficiency prevents macrophage differentiation toward a pro-inflammatory phenotype. Pathway enrichment analysis showed that several biological processes related to inflammation and cell metabolism were modulated by the MR in aged macrophages. Further, transcriptome analysis of aged cardiac fibroblasts revealed that macrophage MR deficiency reduced the activation of pathways related to inflammation and upregulation of ZBTB16, a transcription factor involved in fibrosis. Phenotypic characterization of macrophages showed a progressive replacement of the TIMD4^+^MHC-II^neg/low^ macrophage population by TIMD4^+^MHC-II^int/high^ and TIMD4^–^MHC-II^int/high^ macrophages in the aging heart. By integrating cell sorting and transwell experiments with TIMD4^+^/TIMD4^–^macrophages and fibroblasts from old MR^flox^/MR^LysMCre^ hearts, we showed that the inflammatory crosstalk between TIMD4^–^ macrophages and fibroblasts may imply the macrophage MR and the release of mitochondrial superoxide anions. Macrophage MR deficiency reduced the expansion of the TIMD4^–^ macrophage population and the emergence of fibrotic niches in the aging heart, thereby protecting against cardiac inflammation, fibrosis, and dysfunction. This study highlights the MR as an important mediator of cardiac macrophage inflammaging and age-related fibrotic remodeling.

**Supplementary Information:**

The online version contains supplementary material available at 10.1007/s00395-024-01032-6.

## Introduction

Inflammaging, the gradual and persistent increase in inflammation that occurs during aging, contributes to development of cancer and metabolic disorders and is associated with the pathophysiology of cardiovascular diseases [1, 23, 24]. At a mechanistic level, inflammaging is inextricably intertwined with other molecular hallmarks of cardiovascular aging, encompassing alterations in epigenetics, cellular senescence and mitochondrial dysfunction, impaired macroautophagy, disrupted proteostasis, and genomic instability [1]. Therefore, identifying immune mechanisms and regulatory networks underlying cardiac inflammaging is an important goal for aging research.

Resident macrophages are gatekeepers of tissue homeostasis and integrity and prime cells involved in initiation and regulation of inflammatory responses [5, 61]. Heterogeneous populations of macrophages deriving from embryonic progenitors as well as bone marrow precursor cells co-exist in the heart [6, 42]. Age-dependent alterations in the cardiac microenvironment and intrinsic dysregulations can lead to phenotypic and functional changes in resident macrophages that could have profound effects on cardiac aging [32, 34, 58, 59]. A better understanding of macrophage inflammation in the heart during aging may lead to the development of novel approaches to prevent and/or treat age-related heart diseases such as fibrosis and decline in cardiac function [43, 45]. Gene expression profiling of macrophage populations, isolated from hearts of 15–25 weeks old *Cx*_*3*_*cr1*^*GFP/*+^ mice, revealed early age-dependent alterations in pro-fibrotic genes [48]. Recent single-cell transcriptomics studies showed changes in cellular heterogeneity of cardiac fibroblasts [62] and alterations in myocardial T cells [4] with age. However, our knowledge of the effects of aging on macrophage transcriptome and function in the heart remains quite limited.

Mineralocorticoid receptor (MR) activation promotes inflammation, oxidative stress, obesity-associated coronary microvascular dysfunction, cardiac and arterial fibrotic remodeling and calcification [2, 8, 9, 28, 33]. Experimental studies using cell-selective MR-null or MR-overexpressing transgenic mice emphasized the cell-specific function of the MR in the cardiovascular system and identified the central role of MR signaling in macrophages in inflammatory and fibrotic mechanisms [7, 22, 29, 57]. However, the potential role of the macrophage MR in inflammation and fibrosis in the healthy aging heart has not been defined.

We hypothesized that the MR plays a prominent role in regulating cardiac macrophage inflammaging. To test our hypothesis, we performed transcriptome profiling of cardiac macrophages from male/female MR^flox^ and MR^LysMCre^ mice over the course of aging and showed that the MR promotes macrophage differentiation toward a pro-inflammatory phenotype in the aging heart. Analysis of macrophage heterogeneity and multicolor immunofluorescence staining were integrated and showed that heart aging is characterized by a progressive expansion of the TIMD4^–^ macrophage population, mainly restricted to areas of fibrosis. By integrating macrophage/fibroblast cell sorting and transwell co-culture experiments, we were able to show that the inflammatory crosstalk between TIMD4^–^ macrophages and fibroblasts may involve the macrophage MR and the release of mitochondrial superoxide anions. Macrophage MR deficiency reduced the expansion of the TIMD4^–^ macrophage population in the aging heart and protected against cardiac inflammation and fibrotic remodeling. Moreover, we analyzed the transcriptomes of cardiac fibroblasts from young/old MR^flox^ and MR^LysMCre^ mice and showed that loss of macrophage MR prevented or attenuated inflammatory and osteogenic gene expression in aged fibroblasts. Thus, our findings support that macrophages drive aging processes in the heart and identified the MR as a crucial player in macrophage inflammaging and age-related fibrotic remodeling.

## Materials and methods

### Study protocol

All animal experiments were conducted in accordance with the Guide for the Care and Use of Laboratory Animals published by the National Institutes of Health (Publication No. 85–23, revised 1985). All procedures were approved by the Niedersächsisches Landesamt für Verbraucherschutz und Lebensmittelsicherheit (Oldenburg, Germany; permit No. 33.12-42,502-04-11/0644; 33.9-42,502-04-13/1124 and 33.12-42,502-04-17/2702). Male and female Nr3c2^tm2Gsc^ (MR^flox^), Nr3c2^tm2Gsc^Lyz2^tm1(cre)lfo^/J (MR^LysMCre^) were used in this study [22].

### Isolation of cardiac cells and fluorescence-activated cell sorting

The hearts were perfused for 6 min (3 mL/min) with perfusion buffer (113 mM NaCl, 4.7 mM KCl, 0.6 mM KH_2_PO_4_, 0.6 mM Na_2_HPO_4_, 1.2 mM MgSO_4_, 12 mM NaHCO_3_, 10 mM KHCO_3_, 10 mM HEPES, 30 mM Taurine, 5.5 mM glucose, 10 mM 2,3-Butanedione monoxime), and subsequently digested for 8 min (3 mL/min) with digestion buffer (400 µM calcium chloride and 0.2 mg/mL Liberase™, Roche Diagnostics, in perfusion buffer), using a modified Langendorff perfusion system. The digestion buffer running through the heart was collected into a petri dish which was placed on a heating plate at 37 °C and where the heart was afterwards transferred. The heart tissue was then smoothly pipetted through a sterile low waste syringe several times to obtain a cell suspension. Subsequently, 12 mL of stop buffer [perfusion buffer supplemented with 30% (v/v) heat-inactivated fetal bovine serum (HI-FCS; Gibco, A3840001)] was added to inhibit enzyme activity. The cell suspension was carefully filtered through a 70 µm cell strainer in a 50 mL conical tube, the cell strainer was washed with perfusion buffer and the cell suspension was centrifuged at 600 g for 20 min. The pelleted cells were washed and resuspended in ice-cold FACS-staining buffer (PBS, supplemented with 0.5% bovine serum albumin and 2 mM EDTA). To prevent capping of antibodies on the cell surface and non-specific cell labeling, all steps were performed on ice and protected from light. Cells were pre-incubated with Mouse BD Fc Block™ (1:50; BD Biosciences, 553,141) for 10 min. Subsequently, fluorochrome-conjugated antibodies were added and incubated for 30 min. Finally, the cells were washed with ice-cold FACS-staining buffer. After pre-selection in side scatter (SSC) vs. forward scatter (FSC) dot plot to exclude debris and FSC vs. time-of-flight (ToF) dot plot to discriminate doublets by gating single-cells, cardiac macrophages were identified as CD45^+^/CD11b^+^/CD64^+^/MerTK^+^ cells, whereas heart monocytes as CD64^+^/CD11b^+^/TIMD4^–^/CCR2^+^/MerTK^−^/Ly6C^high^ cells. Cardiac fibroblasts were identified as CD31^−^/TER119^−^/CD45^−^/CD11b^−^/NG2^−^/MEFSK4^+^/PDGFRα^+^. The following antibodies were used: anti-CD45 (clone 30-F11, 1:200, BioLegend); anti-CD11b (clone M1/70, 1:200, eBioscience; clone M1/70, 1:200, BioLegend); anti-CD64 (clone X54-5/7.1, 1:200, BioLegend; clone X54-5/7.1, 1:200, BD Biosciences); anti-MerTK (clone 2B10C42, 1:200, BioLegend); anti-MHC class II (AF6-120.1, 1:200, BioLegend); anti-TIMD4 (clone RMT4-54, 1:200, BioLegend); anti-Ter119 (clone Ter-119, 1:200, BD Biosciences); anti-CCR2 (clone 47,530, 1:250, BD Biosciences); anti-Ly6C (clone AL-21, 1:400, BD Biosciences); anti-CD31 (clone 390, 1:200, BD Biosciences); anti-NG2 (clone REA989, 1:100, Miltenyi Biotec); anti-PDGFRα (clone APA5, 1:100, BioLegend); anti-MEFSK4 (clone mEF-SK4, 1:50, Miltenyi Biotec). FMO controls were included during acquisition for gating analyses to distinguish positive from negative staining cell populations. FACS data were acquired on a Gallios™ flow cytometer and analyzed with Gallios™ software (Beckman Coulter). Cell sorting was performed using a FACS Aria Fusion (BD Biosciences). Cells were sorted in RNeasy Plus lysis buffer (RNeasy Plus Mini Kit; QIAGEN, 74,004) supplemented with 1% β-mercaptoethanol or in RPMI 1640 supplemented with 10% HI-FCS [22].

### RNA-Seq

Total RNA was isolated using RNeasy Mini Kit (QIAGEN) according to the manufacturer’s instructions. Sorted cells were directly collected in RNeasy Plus lysis buffer and immediately processed. RNA quantification and quality testing were assessed by NanoDrop 2000 (Thermo Fisher Scientific) and Bioanalyzer 2100 (Agilent). Libraries for RNA sequencing were prepared from 10 ng total RNA using the NEBNext® Single Cell/Low Input RNA Library Prep Kit (New England BioLabs) according to manufacturer’s protocol. RNA libraries were sequenced using a NovaSeq 6000 system and the NovaSeq 6000 S1 Reagent Kit (100 cycles, paired end run) with an average of 3 * 10^7^ reads per sample (Illumina, San Diego, CA, USA). Prior to aligning with the reference genome, sequences in the raw FASTQ files underwent quality-based trimming and removal of sequencing adapter remnants using fastq-mcf (v1.04.807, http://expressionanalysis.github.io/ea-utils/). Any reads below 15 base pairs were excluded. Trimmed reads were aligned to the reference genome (GCRm39) using the open-source short read aligner STAR (v2.4.2a, https://code.google.com/p/rna-star/), employing settings from the log file. Feature counting was performed using R package Rsubread (v2.10.5) and Ensembl gene annotation file Mus_musculus.GRCm39.109.gtf. Annotation of transcripts was done by R package biomaRt (v2.54.1). Data normalization and differential gene expression analysis were performed using the R package DESeq2 (v1.14.1). Time course analysis of RNA-Seq data was performed with likelihood-ratio test (LRT) implemented in the R package DESeq2. Genes with expression changes over time were identified comparing the design formula ‘age’ against the reduced ‘ ~ 1’. Heatmap was generated with *z* score normalized expression and row-scaled values using R package pheatmap (v1.0.12). Pathway enrichment analysis was performed with GSEA-MSigDB (www.gsea-msigdb.org; m5.go.bp.v2023.1Mn.symbols.gmt) using pre-ranked differentially expressed genes with FDR (false discovery rate) < 0.1; significantly Gene Ontology (GO) terms with *p* < 0.01 were taken for the subsequent analysis and summarized by reducing redundant GO terms using the open access software Revigo (http://revigo.irb.hr/). Pathway enrichment analysis from RNA-seq data in fibroblasts was performed using the function gseKEGG of the package clusterProfiler (v4.0.5) and the annotation data package KEGG.db (v3.2.3) including DEGs (differentially expressed genes) with *p *value less than 0.05. Upstream regulator analysis was performed employing Ingenuity Pathway Analysis Software (IPA, Ingenuity® Systems, www.ingenuity.com).

### RT-qPCR

Reverse transcriptase was performed using 15 ng (macrophages) and 25 ng (fibroblasts) of total RNA and iScript Reverse Transcription Supermix (Bio-Rad) following the manufacturer’s instructions. Relative quantitation of mRNA expression levels was determined with CFX96 Touch Real Time PCR using SsoAdvanced Universal SYBR Green Supermix and PrimePCR Primers (Bio-Rad). Tubulin beta 2A (Tubb2a) was chosen as endogenous control. PCR amplification was performed at initially 95 °C for 30 s followed by 40 cycles at 95 °C for 5 s and terminated by 60 °C for 30 s. The delta-delta Ct method was employed for data analysis.

### Co-culture and assessment of cytokines and pro-collagen levels

For transwell co-culture assays, fibroblasts were plated in 24-well plates and TIMD4^+^/TIMD4^−^ macrophages (45,000–55,000 cells/insert) were seeded in 0.4 μm transwell inserts (Thermo Scientific™, Nunc 140,620) in RPMI 1640 medium supplemented with 2% HI-FCS. After overnight adherence, fibroblasts were co-cultured with TIMD4^+^ and TIMD4^–^ macrophages for 3 days at a ratio of 2 to 1 (fibroblasts: macrophages). For MitoTEMPO experiments, TIMD4^−^ macrophages were incubated in the presence of 25 μM MitoTEMPO (Enzo Life Sciences, ALX430-150-M005) overnight before co-culture with fibroblasts. The levels of IL-6, CCL2, and IL-1ß in the cell-culture supernatants were measured using the LEGENDplex Mouse Inflammation Panel (BioLegend, 740,446). The amount of pro-collagen I alpha 1 in the cell-culture supernatants was determined using a Mouse Pro-collagen I alpha 1 ELISA Kit (Abcam, ab210579).

### Mitochondrial O_2_^·−^ production in TIMD4^+^/TIMD4^–^ macrophages

After 3 days co-culture, the transwell inserts containing macrophages were removed and placed into another 24-well plate. TIMD4^+^ and TIMD4^–^ macrophages were incubated with MitoSOX (2 μM; Invitrogen, M36008) for 30 min. Subsequently, the transwell inserts were washed with RPMI 1640 medium and then placed in 300 μL of ice-cold acetonitrile. The suspension was then transferred into Eppendorf tubes (Safe-Lock, amber; Eppendorf, 0030120.191) and centrifuged at 12,000 g for 10 min at 4 °C. The mitochondria-targeted hydroethidine (2-OH-MitoE^+^) was measured by ion-pair HPLC and electrochemical detection. Isocratic elution was performed (flow rate 0.8 mL/min) using a Synergi 4μ PolarRP80A column (250 × 460 mm; Phenomenex, 00G-4336-E0). The electrochemical detection system consisted of an ESA Coulochem III detector equipped with a Modell 5011 analytical cell (first electrode, 0.00 V; second detecting electrode + 0.30 V). Data acquisition and analysis were performed using the Chromeleon®7 Software (Dionex). Results were normalized for number of cells [22].

### Immunofluorescence

For immunofluorescence, 5-μm cryosections were fixed for 20 min with Cytofix (BD Biosciences, 5,546,555) and permeabilized using 0.1% Triton-X in PBS supplemented with 1% BSA (Miltenyi Biotec, 130-091-376) or cold acetone ( – 20 °C) for 10 min. Subsequently, sections were blocked with 2% donkey serum (LINARIS, LIN-END9010) for 30 min and incubated overnight with primary antibodies, followed by incubation with the secondary antibodies for 60 min at RT in the dark. The M.O.M. (Mouse on Mouse) immunodetection kit (Vector Laboratories, PK-2200) was used for localization of ZBTB16. Antibodies included: anti-ZBTB16 (Santa Cruz Biotechnology, sc-28319); anti-PDGFR alpha (R&D Systems, AF1062); anti-collagen type 1 (Millipore, AB765P); anti IL-1ß (Abcam, ab9722); anti-CCL2 (Abcam, ab308522); anti-TIMD4 (Abcam, ab307558; Novus Biological, NBP1-76,702); donkey anti-goat secondary antibody, Alexa Fluor™ Plus 647 (Invitrogen, A32849); Cy™3 AffiniPure Donkey Anti-Mouse (Jackson ImmunoResearch, 715-165-150); donkey anti-rabbit secondary antibody, Alexa Fluor™ Plus 488 (Invitrogen, A32790); donkey anti-rabbit secondary antibody, Alexa Fluor™ Plus 594 (Invitrogen, A32754). For CD68 immunofluorescence sections were stained with Alexa Fluor 594–labeled (BioLegend, 137,020) or Alexa Fluor 488 (BioLegend, 137,012) anti-CD68. Nuclei were stained with NucBlue™ Live ReadyProbes™ Reagent (Hoechst 33,342; Invitrogen, R37605).

### Cardiac IL-6, hydroxyproline, and collagen content

The hearts were perfused with PBS, and then the right ventricle and left ventricle, including the septum, were separated in ice-cold saline. Left ventricular samples were homogenized in ice-cold RIPA buffer (Cell Signaling Technology, 9806) and centrifuged at 12,000 g for 20 min at 4 °C. Protein concentrations were determined with Bio-Rad Protein Assay Kit II (Bio-Rad, 5,000,006). Levels of IL-6 were measured using the LEGENDplex Mouse Inflammation Panel. Cardiac IL-6 levels were normalized to protein and expressed as pg/mg protein. For hydroxyproline determination, left ventricular tissues were hydrolyzed in 6N HCl at 120 °C for 3 h. Hydroxyproline levels were measured using the Hydroxyproline Assay Kit (Sigma-Aldrich, MAK008). The hydroxyproline content was expressed in μg/mg protein. For quantification of interstitial collagen by picrosirius red polarization microscopy, the heart was arrested in diastole by potassium chloride. Formalin-fixed 5-μm sections were stained with 0.1% sirius red F3B in saturated picric acid and examined using a Nikon ECLIPSE 50i microscope equipped with filters to provide circularly polarized illumination. Tissue images were recorded with a cooled digital camera (DS-5Mc, Nikon) and analyzed using SigmaScan Pro 5.0 image analysis software (Systat Software Inc). Collagen content was expressed as a percentage of the area of each image [22]. Sirius red/fast green staining was performed on 10-µm cryosections using the Sirius Red/Fast Green Staining Kit (Chondrex, 9046).

### Hemodynamic measurements

Left ventricular maximal rate of pressure rise and decline (dP/dt_max_ and dP/dt_min_, respectively), left ventricular systolic and left ventricular diastolic pressure were measured under light isoflurane anesthesia using a conductance catheter (SPR-839; Millar Instruments, 840-8111) advanced into the left ventricle via the right carotid artery. Data were recorded on a data acquisition system (PowerLab 8/30, Chart v5.5.6; ADInstruments) [22].

### Statistical analysis

Results are reported as mean ± SEM. One-way ANOVA with Tukey post hoc test or the unpaired *t* test was performed using GraphPad Prism 6.01 (GraphPad Software, Inc). Values of *p* < 0.05 were considered statistically significant.

## Results

### Mineralocorticoid receptor deficiency protects against macrophage inflammaging

**Fig. 1:**
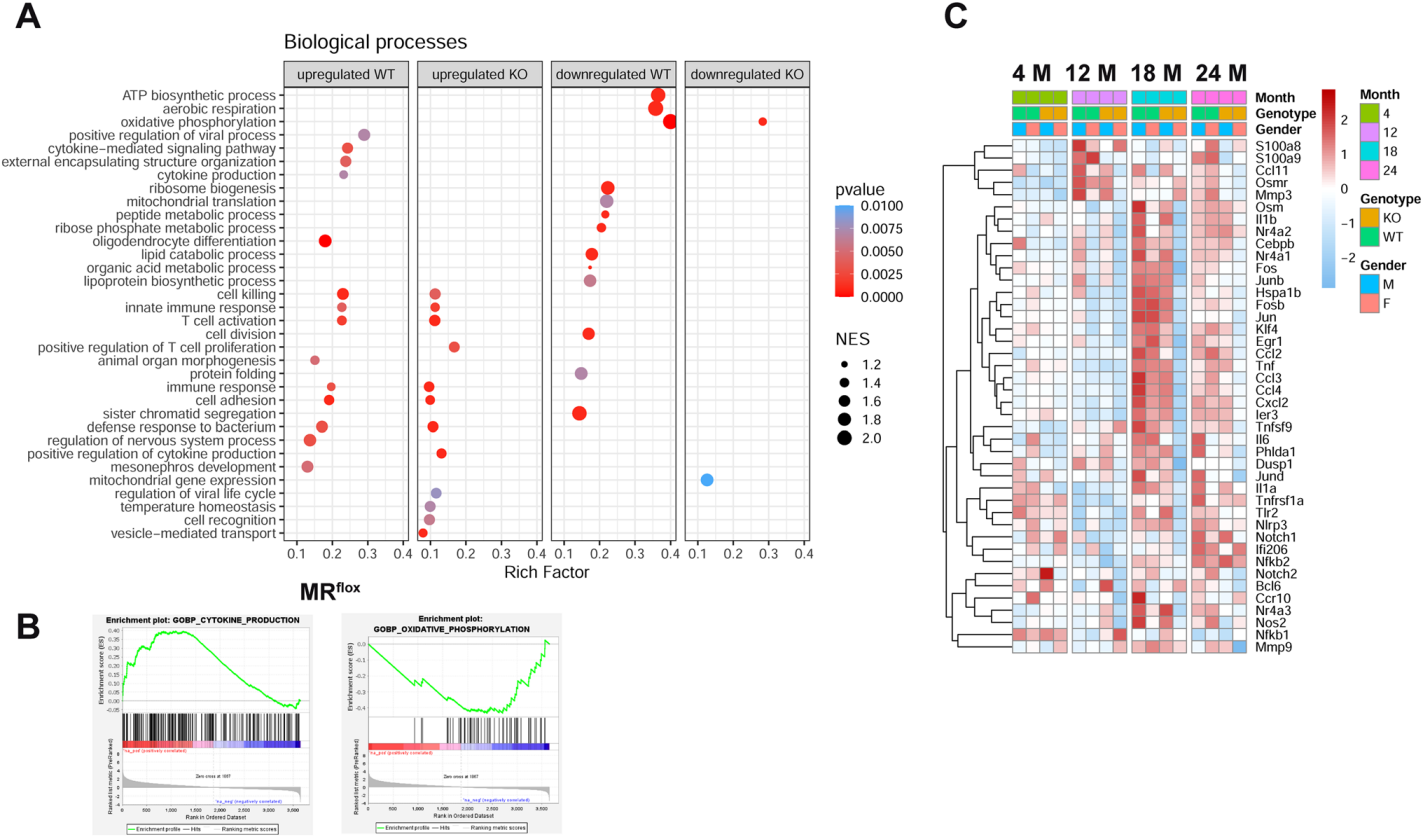
MR deficiency protects against macrophage inflammaging. Cardiac macrophages, identified as CD45^+^/CD11b^+^/CD64^+^ and MERTK^+^, were FACS sorted from hearts of male/female young (4 months-old), middle-aged (12 months-old), and old (18 and 24 months-old) MR^flox^ and MR^LysMCre^ mice. **A** Comparison of biological processes significantly altered (*p* < 0.01) over time in macrophages between MR^flox^ (WT) and MR^LysMCre^ (KO) mice determined by gene set enrichment analysis (GSEA). Parent terms following redundant Gene Ontology terms reduction are shown, except for some child terms when the relative parent terms did not appear in both genotypes. Rich factor is the ratio of differentially expressed gene number (Size) annotated in a pathway term to all gene number annotated. Greater rich factor means greater intensiveness. NES: normalized enrichment score determined by GSEA. **B** GSEA plots of upregulated cytokine production and downregulated oxidative phosphorylation pathways in macrophages from MR^flox^ mice.** C** Heatmap showing *z *score normalized expression values of selected genes over the course of aging in the range 4 to 24 months related to inflammatory macrophages from MR^flox^ (WT) and MR^LysMCre^ (KO) mice reported on published single-cells RNA-seq data or belonging to leading edges of GSEA

Quantitative RT-PCR showed downregulation of Nr3c2 expression in macrophages isolated by cell sorting from the hearts of young and old MR^LysMCre^ mice compared to their MR^flox^ littermates (Supplementary Fig. S1). Likelihood-ratio test (LRT) was used to reveal DEGs over course of aging in cardiac macrophages isolated by cell sorting from hearts of MR^flox^ and MR^LysMcre^ mice (Supplementary Fig. S2). Bioinformatic analysis by LRT reported 9.5% upregulated and 9% downregulated genes for MR^flox^ mice whereas 4.6% upregulated and 2.9% downregulated genes for MR^LysMcre^ mice over the baseline with adjusted *p *value < 0.1 out of 20,122 filtered nonzero reads counts (Supplementary Fig. S2). Pathway enrichment analysis (Fig. 1A, B) showed that several biological processes related to inflammation and cell metabolism and involved in cardiovascular aging [21, 32, 38, 52, 61] were regulated. Of note, over course of aging signaling pathways related to regulation of inflammation as cytokine-mediated signaling pathway, cytokine production, immune response, T cell activation were upregulated while pathways related to cell metabolism as lipid catabolic process, peptide metabolic process, ATP biosynthetic process, organic acid metabolic process were downregulated in macrophages from hearts of MR^flox^ mice. (Fig. 1A, Suppl. Table 1). In contrast, in cardiac macrophages lacking the MR, the altered pathways identified by pathway enrichment analysis over course of aging showed a lower rich factor, indicating reduced intensiveness or did not reach significance (Fig. 1A). A heatmap of selected genes, derived from published single-cell RNA-seq data [15, 34, 51] and genes derived from leading edges of gene set enrichment analysis (GSEA) performed in this study, showed an upregulation of key inflammatory genes (S100a9, Osmr, Ccl11, Mmp3, Klf4, Fos, Jun, Junb, Tnfrsf1a, Egr1, Il6, Il1a, Il1b, Notch1/2, Nos2, Ccl2, Nr4a1, Nr4a3, and Nlrp3) in cardiac macrophages from MR^flox^ mice at 18–24 months (Fig. 1C). Myeloid cell-restricted MR deficiency attenuated or prevented the deregulation of factors involved in inflammation in aging cardiac macrophages (Fig. 1C). Of interest, the prevention of macrophage polarization to a pro-inflammatory phenotype was shared between the sexes but more marked in female MR^LysMcre^ mice (Fig. 1C).

### Macrophage mineralocorticoid receptor deficiency affects the transcriptome of fibroblasts in the aging heart

**Fig. 2:**
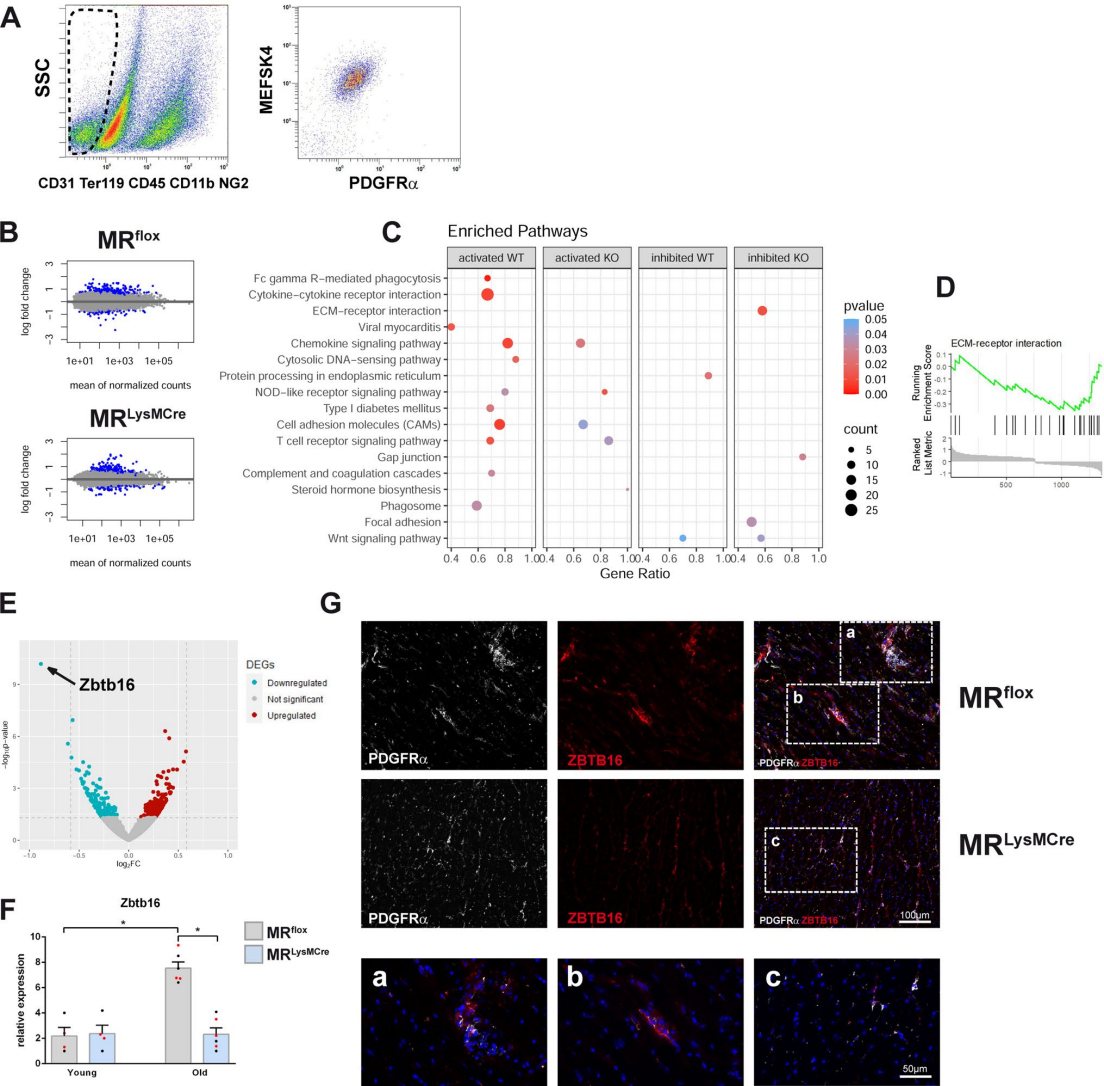
Macrophage MR deficiency affects the transcriptome of fibroblasts in the aging heart. **A** Gating strategy to identify CD31^−^/TER119^−^/CD45^−^/CD11b^−^/NG2^−^/MEFSK4^+^/PDGFRα^+^ cells. Fibroblasts were isolated by flow cytometry from hearts of male/female young and old MR^flox^ and MR^LysMcre^ mice. **B** MA plots of gene expression in fibroblasts. Significantly differentially expressed genes (old vs. young) with adjusted *p* values < 0.1 are indicated in blue. **C** Graphical dot plot representation of the most significantly enriched pathways in fibroblasts. Comparison of altered pathways (*p* < 0.01) between MR^flox^ (WT) and MR^LysMCre^ (KO) mice determined by R package clusterProfiler. Large gene ratio in combination with high count and low *p *value indicates a more pronounced activation or inhibition for a specific pathway; **D** GSEA plot of downregulated ECM–receptor interaction pathway in fibroblasts from MR^LysMCre^ mice. **E** Volcano plot of contrast of old cardiac fibroblasts from MR^LysMcre^ vs. MR^flox^ mice. **F** Relative expression of Zbtb16 in young/old fibroblasts from MR^flox^ and MR^LysMCre^ hearts. Depicted points in red/black indicate, respectively, male and female mice. **G** Immunofluorescence micrographs of heart sections from old MR^flox^ and MR^LysMCre^ mice showing fibroblasts (PDGFRα^+^ cells) and ZBTB16 immunoreactivity. MR^LysMCre^ hearts displayed a weak expression of ZBTB16 in PDGFRα-positive fibroblasts. Nuclei were stained with NucBlue™. Mean ± SEM, *n* = 4–6 per group; *p < 0.05

We previously showed that dynamic interactions between macrophages and fibroblasts are critically regulated by the macrophage MR during myocardial infarct healing [22]. To expand our understanding of potential mechanisms underlying protection via MR deficiency in macrophages against cardiac aging, we investigated the impact of myeloid cell-specific MR deficiency on aging-induced gene expression changes in cardiac fibroblasts. We performed gene expression profiling of fibroblasts isolated by cell sorting from hearts of male/female young (3 ± 0.5 months old) and old (20 ± 1 months old) MR^flox^ and MR^LysMcre^ mice (Fig. 2A–D). Bioinformatic analysis revealed 1.48% and 1.46% deregulated genes with adjusted *p* value < 0.1 in fibroblasts from MR^flox^ and MR^LysMcre^ old hearts, respectively, compared to young fibroblasts (Fig. 2B). Pathway analysis revealed that fibroblasts from hearts of old MR^flox^ mice were enriched in cytokine–cytokine receptor interaction, chemokine signaling, complement and coagulation cascades, and phagosome pathways (Fig. 2C). Macrophage MR deficiency reduced or prevented the activation of the transcriptional pathways related to inflammation in aged cardiac fibroblasts (Fig. 2C). Of interest, the ECM–receptor interaction pathway, including several genes present in the core enrichment, like collagen genes (Col1a1, Col1a2, Col3a1, Col5a2, Col6a2, Col11a1), was significantly inhibited in cardiac fibroblasts from MR^LysMCre^ mice (Fig. 2D). Moreover, using IPA, we performed upstream regulator analysis to identify potential cytokines/inflammatory regulators that may be involved in the modulation of the transcriptional profile of aged cardiac fibroblasts by MR deficiency in macrophages (Supplementary Fig. S3A). We found that Il6 and Il1b were predicted to be inhibited upstream regulators (Supplementary Fig. S3B). In addition, we identified ZBTB16 (transcriptional suppressor zinc finger and BTB domain containing 16) as the strongest downregulated gene in old cardiac fibroblasts from MR^LysMcre^ mice vs. MR^flox^ (Fig. 2E). Quantitative PCR showed that the expression of ZBTB16 was upregulated in aging fibroblasts compared to respective young fibroblasts and significantly lower in fibroblasts from both male and female old MR^LysMCre^ mice (Fig. 2F). In old hearts from MR^flox^ mice, we found a strong immunoreactivity for ZBTB16 in PDGFRα-positive cells in areas of fibrosis (Fig. 2G), whereas old hearts of MR^LysMCre^ mice displayed a weak expression of ZBTB16 in PDGFRα-positive fibroblasts (Fig. 2G). Thus, these data suggest a role for macrophage MR signaling in regulating fibroblast polarization during aging as well as corroborated a recent single-nucleus RNA-sequencing study showing that aged fibroblasts exhibited changed expression patterns of inflammatory and osteogenic genes [62].

### Macrophage MR deficiency reduced the expansion of the TIMD4^–^ macrophage population in the aging heart

**Fig. 3:**
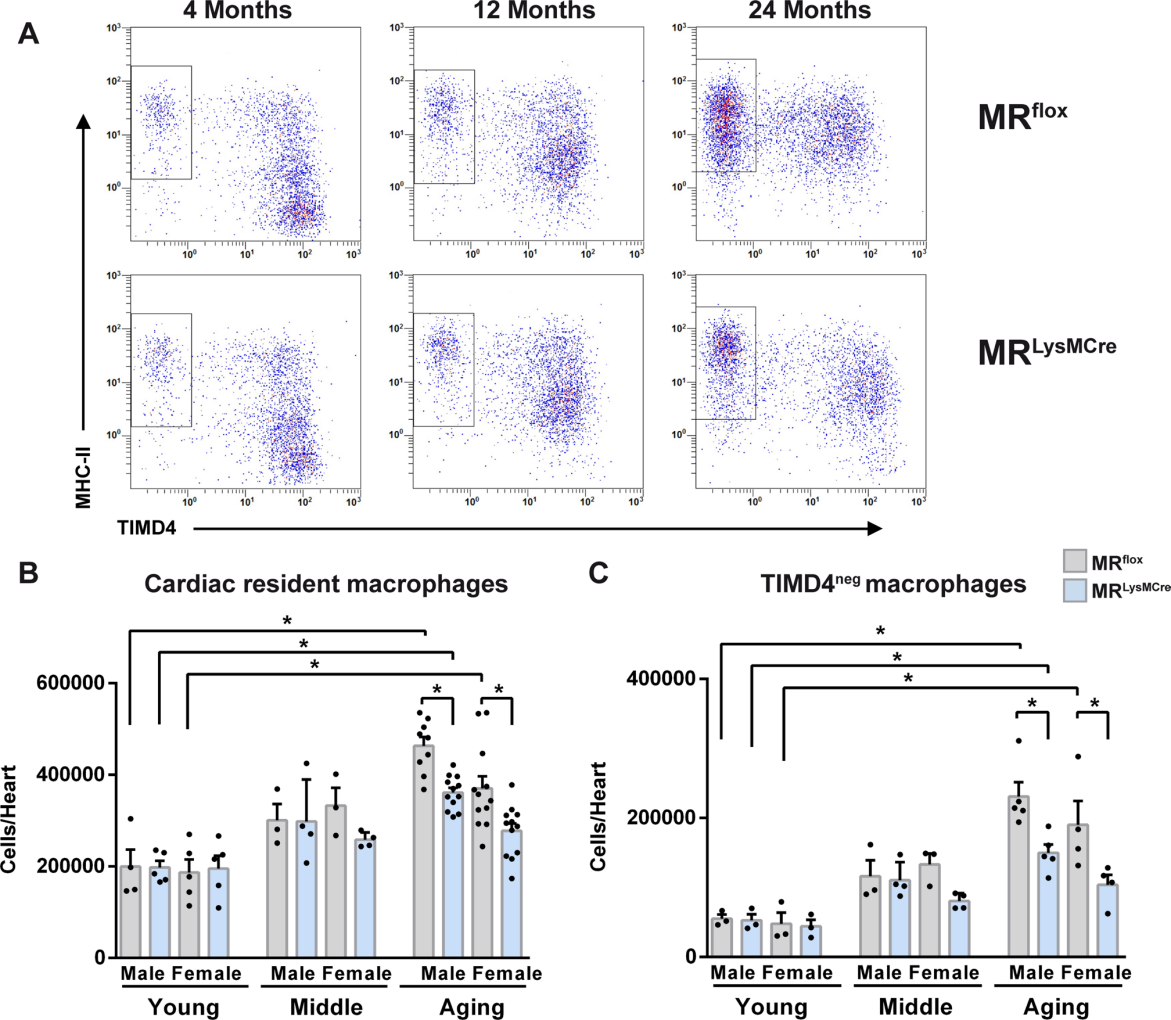
Macrophage MR deficiency reduced the expansion of the TIMD4^–^ macrophage population in the healthy aging heart. **A** Flow cytometric analysis of macrophages from hearts of young, middle-aged, and old MR^flox^ and MR^LysMCre^ mice. Cardiac macrophages were identified as positive for CD45, CD11b, CD64 and stratified by MHC-II and TIMD4. FACS-based quantification of **B** cardiac resident macrophages and **C** TIMD4^–^ macrophages. Mean ± SEM, *n* = 3–12 per group; *p < 0.05

The heart contains heterogeneous subsets of resident macrophages [6, 16, 17, 42]. Having discovered that MR deletion prevents macrophage polarization toward a pro-inflammatory phenotype in the aging heart, we next determined whether there were associated changes in the number and proportion of macrophage subsets. Macrophage heterogeneity in the heart was investigated by flow cytometry in young (3 ± 0.3 months old), middle-aged (12 ± 0.4 months old), and old (21 ± 0.4 months old) MR^flox^ and MR^LysMCre^ mice (Fig. 3; Supplementary Fig. S4). Cardiac macrophages were identified as positive for CD45, CD11b, CD64 and divided into subpopulations based on the surface expression of major histocompatibility complex II (MHC-II), TIMD4 and CCR2 (Fig. 3A; Supplementary Fig. S4A) [16, 17, 46]. The number of cardiac macrophages was increased in old male/female MR^flox^ and male MR^LysMCre^ mice compared to respective young mice (Fig. 3B) and was significantly lower in both male and female old MR^LysMCre^ mice as compared to old MR^flox^ (Fig. 3B). In young mice, the majority of macrophages was TIMD4^+^ MHC-II^neg/low^ (Fig. 3A). Interestingly, TIMD4^+^ macrophages upregulated MHC-II expression over time, and with age there was a progressive increase in TIMD4^–^MHC-II^int/high^ macrophages in old male/female MR^flox^ and male MR^LysMCre^ mice (Fig. 3A, C). The amount of TIMD4^–^MHC-II^int/high^ macrophages was significantly lower in both male and female old MR^LysMCre^ mice as compared to old MR^flox^ mice (Fig. 3C). Dick et al., performing sequential long-term parabiotic studies, provided evidence that while cardiac TIMD4^–^ macrophages expressing CCR2 were continually replaced by blood monocytes in adult mice (6 or 10 months old), TIMD4^–^CCR2^–^ macrophages had an intermediate level of replacement that remained stable between 6 and 10 months [17]. We found a significant expansion of the TIMD4^–^CCR2^+^ and TIMD4^–^CCR2^–^ macrophage subsets in old male/female MR^flox^ and male MR^LysMCre^ mice compared to respective young mice, which was significantly lower in both male and female old MR^LysMCre^ mice vs. old MR^flox^ (Supplementary Fig. S4). Hence, the macrophage MR plays a crucial role in the recruitment of monocyte-derived macrophages in the heart during aging. Interestingly, Nr3c2 expression levels were not significantly different in TIMD4^+^ and TIMD4^–^ macrophages isolated from the hearts of young and old mice (Supplementary Fig. S1B). A significant downregulation of Nr3C2 expression was found in young/aged TIMD4^+^/TIMD4^–^ macrophages from MR^LysMCre^ mice compared to MR^flox^ (Supplementary Fig. S1B), further confirming genetic deletion of MR in cardiac macrophages. Heart monocytes can be distinguished from cardiac macrophages based on their expression of MerTK. As shown in Supplementary Fig. S5, CD64^+^/CD11b^+^/TIMD4^–^ cells expressing high levels of Ly6C and CCR2^+^/MerTK^–^ were nearly absent in aged hearts of MR^flox^ and MR^LysMCre^ mice. Overall, these findings confirmed and expanded previous studies which showed the emergence of a macrophage population expressing the macrophage marker Mrc1 and low levels of Cx3cr1 [48], enhanced accumulation of myeloid cells [19], macrophages [32] and lymphocytes [50] in the heart during aging.

### Aged TIMD4^–^ macrophages co-cultured with fibroblasts promote fibrogenic factors production in a MR-dependent manner

Next, we focused on exploring the mechanisms responsible for the reduced expansion of the TIMD4^–^ macrophage population in old MR^LysMCre^ hearts and the functional role of TIMD4^–^ macrophages in driving cardiac inflammaging and fibrosis. We supposed that the dynamic interplay between fibroblasts and different macrophage populations could have significant implications for dysregulated fibrotic responses in the context of aging. Thus, the interactions between cardiac TIMD4^+^/TIMD4^–^ macrophages and fibroblasts and the effect of macrophage MR deficiency were investigated.

**Fig. 4:**
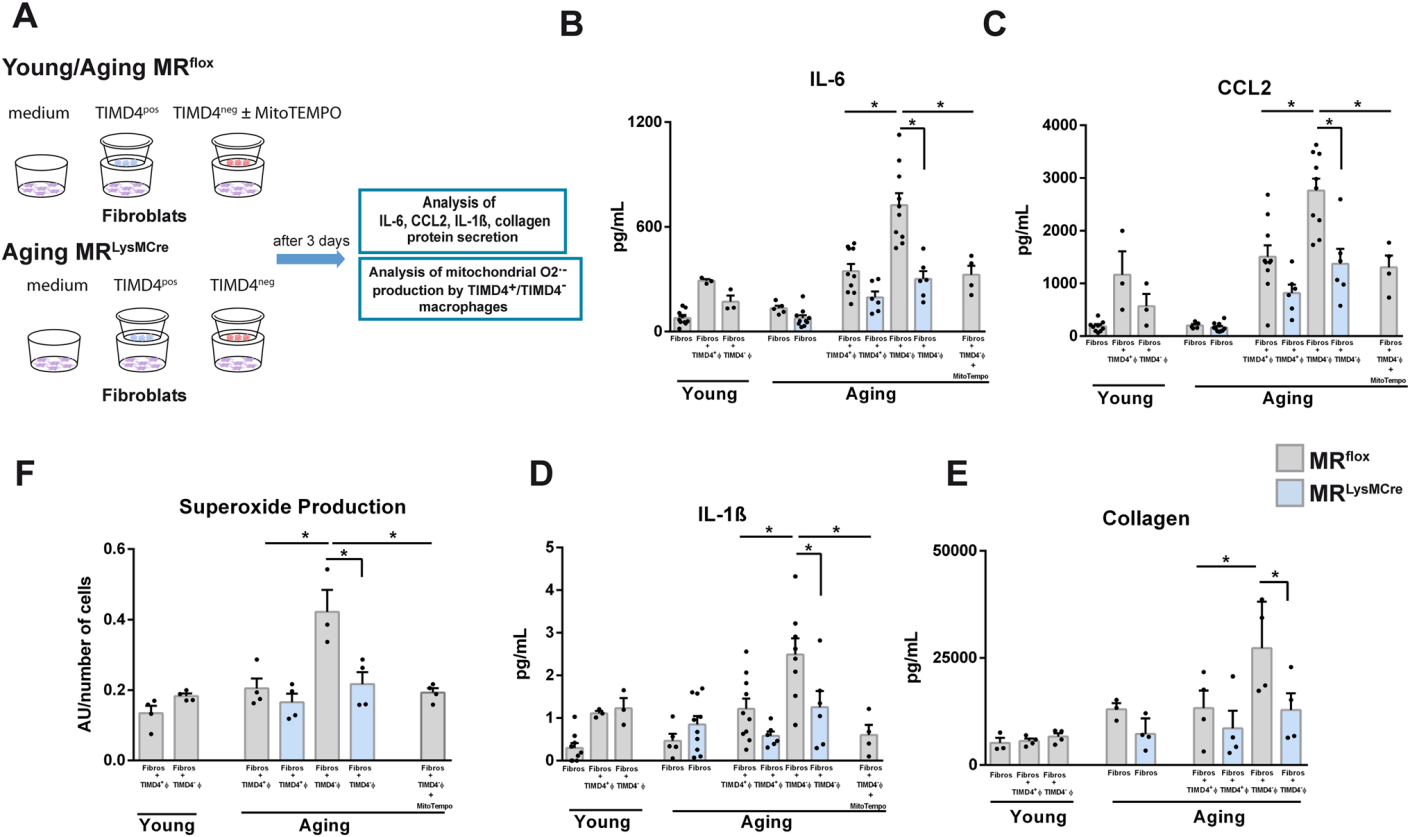
Inflammatory crosstalk between aged TIMD4^–^ macrophages and fibroblasts implies the macrophage MR and superoxide production in the mitochondria. **A** Experimental outline. Cardiac TIMD4^+^ and TIMD4^–^ macrophages and fibroblasts were isolated from hearts of young MR^flox^ mice (Young) or from hearts of old MR^flox^ and MR^LysMCre^ mice (Aging). Macrophage subpopulations were co-cultured with fibroblasts for 3 days. For MitoTEMPO experiments TIMD4^–^ macrophages from aging MR^flox^ hearts were incubated in the presence of MitoTEMPO overnight before co-culture with fibroblasts. **B** IL-6, **C** CCL2, **D** IL-1ß, and **E** pro-collagen I alpha 1 levels in the conditioned medium of cardiac fibroblasts cultured alone or co-cultured for 3 days with TIMD4^+^ and TIMD4^–^ macrophages. **F** Mitochondrial O_2_^·−^ production by TIMD4^+^ and TIMD4^–^ macrophages cultured in the presence of fibroblasts assessed using a highly sensitive isocratic ion-pair HPLC-EC method. The treatment with the mitochondrial reactive oxygen species scavenger MitoTEMPO prevented the production of O2^·−^ by TIMD4^–^ macrophages cultured in the presence of fibroblasts, and secretion of inflammatory cytokines in the co-culture medium of TIMD4^–^ macrophages and fibroblasts isolated from old MR^flox^ hearts. Mean ± SEM, *n* = 3–10 per group; *p < 0.05

To more closely mimic the complexity of the in vivo cardiac environment and to ensure the maintenance of the cell phenotype, we performed transwell co-culture experiments using FACS-sorted cells from hearts of young (3 ± 0.5 months old) MR^flox^ and old (20 ± 0.8 months old) MR^flox^/MR^LysMCre^ male/female mice (Fig. 4A–F). Preliminary experiments showed that current protocols for tissue dissociation involving mincing, mechanical dissociation, and long enzymatic digestion times resulted in cell damage/loss and low recovery of resident macrophages and often required pooling of hearts. Retrograde perfusion with a modified Langendorff system for 6 min to remove blood cells and subsequently digestion for 8 min resulted in isolation of a higher number of macrophages and allowed us to perform transwell experiments using cardiac macrophages instead of (thioglycollate-elicited) peritoneal macrophages/bone marrow-derived macrophages that are commonly used as a surrogate for cardiac macrophages for in vitro experiments (Fig. 4A). Moreover, we isolated resident cardiac fibroblasts by FACS sorting, an approach that has allowed us to obtain highly purified cells, avoiding significant artefacts of conventional culturing and passaging (Fig. 2A).

We found that aged TIMD4^+^/TIMD4^–^ macrophages in culture alone, regardless of their genotype, secreted barely detectable levels of IL-6, CCL2, IL-1ß, and collagen. In contrast, we discovered significantly increased IL-6, CCL2, IL-1ß, and collagen protein levels in the co-culture medium of TIMD4^–^ macrophages and fibroblasts isolated from old MR^flox^ hearts vs. old TIMD4^+^ macrophages/fibroblasts co-cultures (Fig. 4B–E). Notably, a significant increase in inflammatory and collagen proteins was not induced by co-culture of TIMD4^–^ macrophages and fibroblasts isolated from old MR^LysMCre^ and young MR^flox^ hearts. (Fig. 4B–E). To discriminate between the potential contributions of TIMD4^–^ macrophages or fibroblasts to the total secreted IL-6, CCL2, IL-1ß, and collagen protein levels, RNA was extracted from co-cultured cells and analyzed by absolute quantitative PCR (Supplementary Fig. S6). Il1b mRNA was detectable only in TIMD4^–^ macrophages whereas Ccl2 expression was similar in TIMD4^–^macrophages and fibroblasts. The expression of Il6 and Col1α2 was 20.7- and 50.5-fold higher, respectively, in fibroblasts than TIMD4^–^ macrophages (Supplementary Fig. S6B). Overall, fibroblasts drove aged TIMD4^–^ macrophages to secrete IL-1ß and CCL2 and TIMD4^–^ macrophages stimulated fibroblasts to produce CCL2, IL-6, and collagen, through a contact-independent mechanism. Next, we sought to characterize the potential mediators involved in the pro-fibrotic crosstalk between TIMD4^–^ macrophages and fibroblasts.

### Suppression of mitochondrial oxidative stress in aged TIMD4^–^ macrophages lacking the MR cultured in the presence of fibroblasts

Mitochondrial oxidative stress plays a critical role in cardiovascular aging, and cellular damage by oxygen free radicals has been implicated as a driving force for cardiac inflammaging [53]. Accordingly, we next assessed whether superoxide anions produced in the mitochondria of macrophages are involved in the enhanced production of inflammatory cytokines in our transwell co-culture experiments (Fig. 4A). Mitochondrial O_2_^·−^ production in TIMD4^+^/TIMD4^–^ macrophages was determined using a highly sensitive isocratic ion-pair HPLC-EC method to selectively detect the superoxide-specific product of mitochondria-targeted hydroethidine (2-OH-MitoE^+^). As shown in Fig. 4F, mitochondrial superoxide anion levels were significantly higher in TIMD4^–^ macrophages isolated from aged MR^flox^ mice after 3 days co-culture with fibroblasts. Enhanced generation of superoxide anions (Fig. 4F) by TIMD4^–^ macrophages as well as IL-6, CCL2, and IL-1ß secretion in the co-culture medium (Fig. 4B–D) were similarly prevented by deficiency of the MR and the treatment with the mitochondria-targeted antioxidant MitoTEMPO. Collectively, by integrating cell sorting and transwell co-culture experiments, we were able to demonstrate that the inflammatory crosstalk between TIMD4^–^ macrophages and fibroblasts implies the release of mitochondrial superoxide anions and the macrophage MR.

### MR deficiency in macrophages protects mice from cardiac inflammation, fibrosis, and heart dysfunction induced by aging

**Fig. 5:**
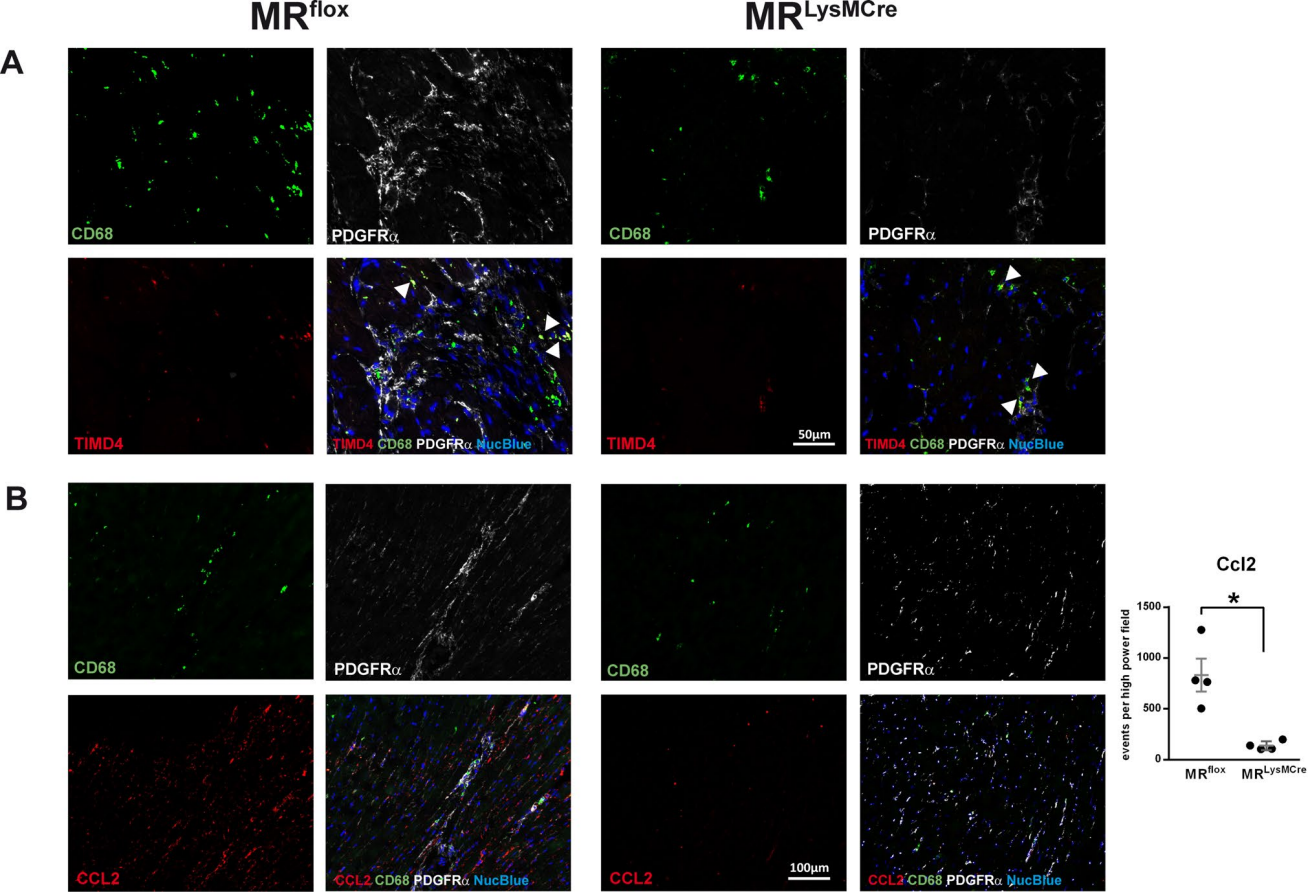
Immunofluorescence micrographs of heart sections from old MR^flox^ and MR^LysMCre^ mice showing **A** macrophages (CD68^+^ cells), fibroblasts (PDGFRα^+^ cells), and TIMD4 immunoreactivity. Arrows indicate CD68^+^ and TIMD4^+^ cells. In the aged heart of MR^flox^ mice, TIMD4^–^ macrophages were localized predominantly to areas containing a large amount of fibroblasts. **B** Immunoreactivity for CCL2 in CD68 and PDGFRα-positive areas. Old hearts from MR^LysMCre^ mice displayed a weak expression of CCL2 in PDGFRα-positive areas. Nuclei were stained with NucBlue™. Mean ± SEM, *n* = 4 per group; *p < 0.05

**Fig. 6:**
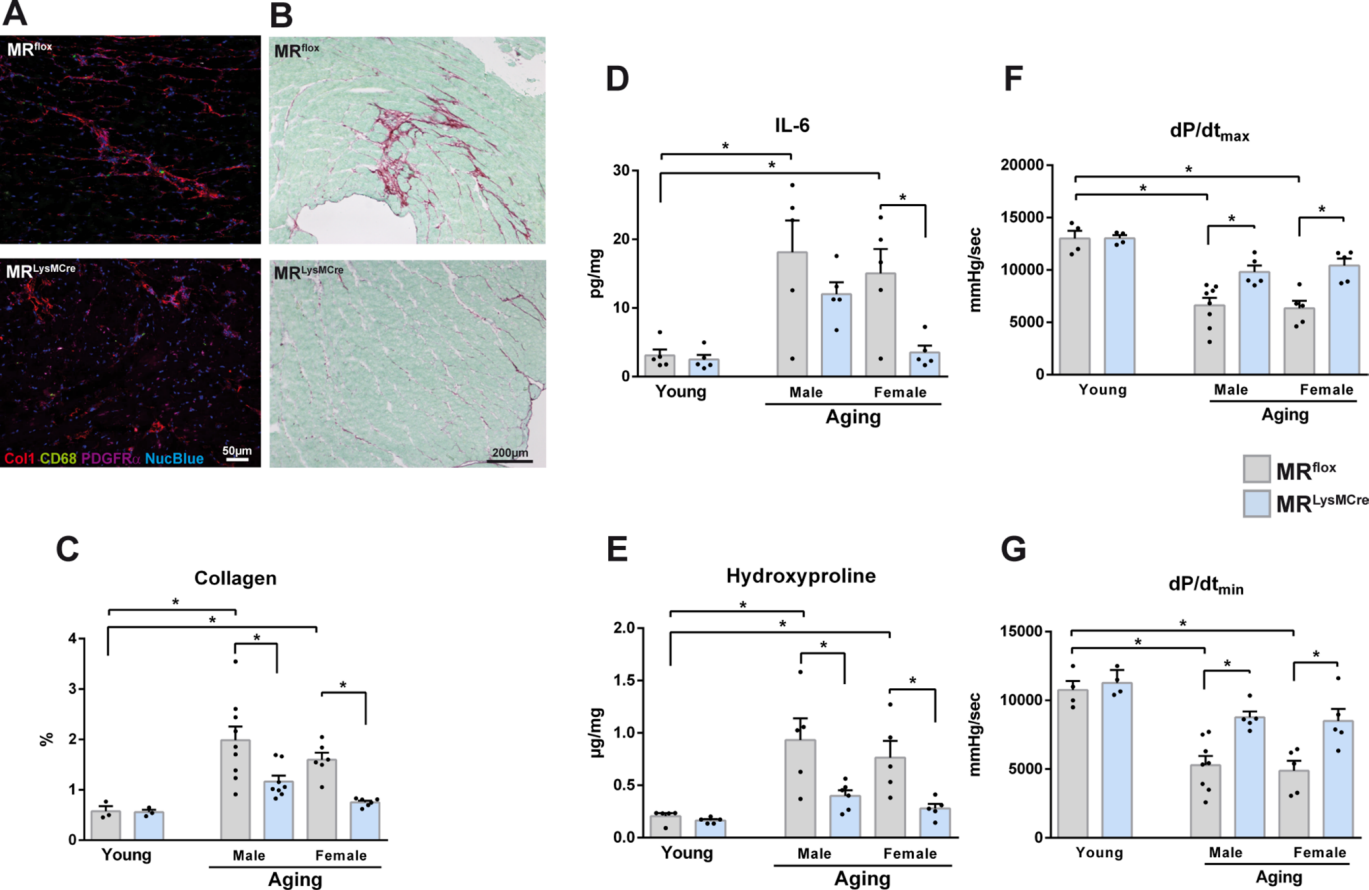
MR deficiency in macrophages protects mice from cardiac inflammation, fibrosis, and heart dysfunction induced by aging. **A** Immunofluorescence micrographs of heart sections from old MR^flox^ and MR^LysMCre^ mice showing macrophages (CD68^+^ cells), fibroblasts (PDGFRα^+^ cells), and collagen type 1 immunoreactivity. **B** Sirius red/fast green staining of representative heart sections from old MR^flox^ and MR^LysMCre^ mice. **C** Interstitial fibrosis quantified by picrosirius red polarization microscopy, **D** cardiac IL-6 protein and **E** hydroxyproline levels in young and old MR^flox^ and MR^LysMCre^ mice. **F** Left ventricular maximal rate of pressure rise (dP/dt_max_) and **G** maximal rate of pressure decline (dP/dt_min_) measured in vivo with a conductance catheter in young and old MR^flox^ and MR^LysMCre^ mice. Aged MR^flox^ and MR^LysMCre^ mice were on average 22 (± 0.7) months old. Mean ± SEM, *n* = 3–9 per group; *p < 0.05

To establish the relevance of our in vitro findings to in vivo situation, next, we defined TIMD4^–^ macrophages localization (Fig. 5A) and evaluated CCL2 (Fig. 5B) and collagen type I (Fig. 6A) immunoreactivity in the aging heart. In old hearts, immunofluorescence revealed that TIMD4^–^ macrophages (CD68^+^ cells) were localized predominantly to fibrotic areas containing a large amount of PDGFRα-positive cells (Fig. 5A). Likewise, strong immunoreactivity for CCL2 was restricted to regions with a high density of fibroblasts (Fig. 5B). In the hearts of young MR^flox^/MR^LysMCre^ mice (Supplementary Fig. S7A), TIMD4^–^CD68^+^ cells were barely detectable. The hearts of old MR^LysMCre^ and young MR^flox^/MR^LysMCre^ mice displayed a weak expression of CCL2 in PDGFRα-positive areas (Fig. 5B; Supplementary Fig. S7B). Moreover, old MR^LysMCre^ hearts showed lower collagen type I immunoreactivity (Fig. 6A) and macrophages expressing IL-1ß were hardly detected in the aged heart of MR^LysMCre^ mice (Supplementary Fig. S8). These data suggest that dynamic interactions between macrophages and fibroblasts involving the macrophage MR may regulate the expansion of fibrotic areas in the aged heart.

**Fig. 7:**
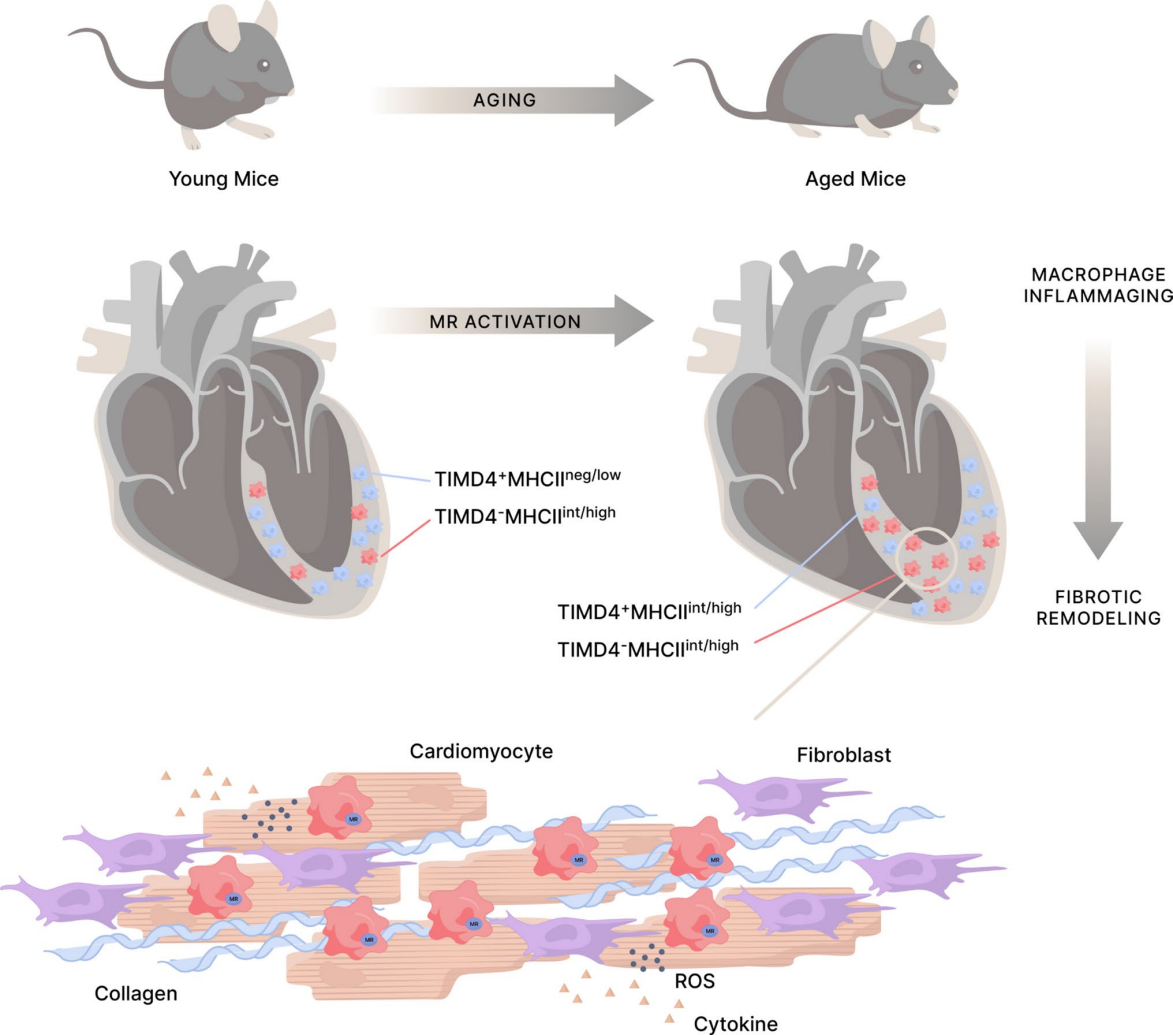
Mineralocorticoid receptor activation in macrophages during healthy aging leads to dysregulation of biological processes related to inflammation and cell metabolism, which promotes cardiac macrophage inflammaging. Heart aging is characterized by a progressive expansion of the TIMD4^–^ macrophage population that mediates inflammatory and fibrogenic responses. TIMD4^–^ macrophages are mainly restricted to areas of fibrosis, containing a large amount of fibroblasts. The inflammatory crosstalk between TIMD4^–^ macrophages and fibroblasts implies the macrophage MR and the release of mitochondrial superoxide anions. MR deficiency in macrophages prevents the activation of pathways related to inflammation in fibroblasts, reduces the expansion of the TIMD4^–^ macrophage population and the emergence of fibrotic niches in the aging heart, thereby protecting against cardiac inflammation and fibrotic remodeling

Further, we found that the age-associated increase in IL-6 levels in the left ventricular tissues of old MR^flox^ mice tended to be lower in age-matched male MR^LysMCre^ mice and was significantly prevented in female MR^LysMCre^ mice (Fig. 6D). In addition, we quantified cardiac fibrosis by picrosirius red polarization microscopy. We revealed significantly increased interstitial fibrosis in the myocardium of old MR^flox^ mice (Fig. 6A–C), in both male and female with more pronounced increase in males. Increased cardiac fibrosis in aging was further confirmed by measurement of hydroxyproline content (Fig. 6E). Enhanced interstitial fibrosis and hydroxyproline levels were inhibited in the age-matched male and female MR^LysMCre^ mice (Fig. 6B, C, E). The progressive accumulation of fibrotic tissue in the aging myocardium can lead to impaired heart function. We did not detect significant differences between young/old MR^flox^ and MR^LysMCre^ mice regarding left ventricular systolic or diastolic pressure (Supplementary Fig. S9). We found that aging in MR^flox^ mice was associated with decreased dP/dt_max_ and dP/dt_min_ (Fig. 6F, G), indicating reduction of systolic and diastolic performance. MR deficiency in myeloid cells was sufficient to elicit protection against age-associated decline in cardiac function in male and female mice (Fig. 6F, G). Altogether, the presence of the MR promotes a macrophage phenotype that mediate fibrosis, inflammaging, and pump dysfunction in the aging heart (Fig. 7).

## Discussion

Progressive aging triggers several cellular and molecular alterations giving rise to a pro-inflammatory state referred to as inflammaging. In recent years, a growing body of evidence has linked inflammaging to the pathophysiology of cardiovascular aging. This study identifies the MR on macrophages as an important mediator of the inflammaging process in the heart and a potential target to prevent age-related fibrotic remodeling and cardiac dysfunction. Specifically, we showed that macrophage MR deficiency protects against age-dependent pro-inflammatory polarization of cardiac macrophages, attenuates or prevents the activation of pathways related to inflammation in aged fibroblasts and reduces the expansion of the TIMD4^–^ macrophage population that mediates inflammatory and fibrogenic responses in a MR-dependent manner.

In this study, incisive bioinformatic analysis was performed to screen for the key genes and pathways involved in macrophage inflammaging and to reveal novel mechanistic insights into the role of the MR in aging macrophages. We found that macrophage differentiation in the heart over course of aging did not fit straight within a M1/M2 paradigm, but instead demonstrated mixed phenotypes with deregulation of factors and pathways related to regulation of inflammation and cell metabolism. Macrophage MR deficiency blunted macrophage polarization to a pro-inflammatory phenotype and the deregulation of critical actors in the inflammaging process such as NLRP3 inflammasome and the NR4A nuclear receptors [1, 23, 24, 61]. Previous studies have showed that alterations in metabolism induced by NR4A nuclear receptors dysregulation may contribute to inflammation, tumorigenesis, diabetes, and atherosclerosis [66]. Interestingly, the orphan nuclear receptors NR4A subfamily have already been hypothesized as potential targets for geroprotective interventions.

Our mechanistic studies investigating the transcriptome of cardiac fibroblasts from MR^flox^ and MR^LysMCre^ mice suggest that microenvironmentally driven transcriptional modulation of fibroblast polarization in the aging heart is related to MR activation in macrophages. Intriguingly, the MR target gene ZBTB16 was found to be the strongest downregulated DEG in aged fibroblasts by macrophage MR deficiency. ZBTB16, a transcription factor and epigenetic regulator involved in protein–protein interactions, cell proliferation, and differentiation, has been associated with oxidative stress, metabolic syndrome, and fibrosis [54, 60]. Regulation of ZBTB16 expression is complex and can be modulated by several signaling pathways involved in cellular stress responses, including oxidative stress and inflammation [54]. Notably, deficiency of MR in smooth muscle cells has recently been shown to prevent western diet-induced inflammation across the cardiac cellulome and the upregulation of ZBTB16 in cardiac fibroblasts [18]. Dysregulation of ZBTB16 has been previously linked to aging in healthy human individuals [25]. A recent study showed that chronic stress-driven glucocorticoid receptor activation induces strong ZBTB16 expression along with functional epigenomic changes in human fibroblasts [39]. Upregulation of ZBTB16 has been implicated in cardiac fibrosis and hypertrophy in the spontaneously hypertensive rat [40] and in response to angiotensin II [56, 63]. Furthermore, ZBTB16 promotes osteogenic differentiation and aortic valve calcification [3, 55]. Thus, the induction of ZBTB16 in aging fibroblasts may contribute to increased calcification in the aging heart [62]. However, the potential role of ZBTB16 in inflammaging and whether ZBTB16 being causative for the aging process in the heart definitely requires further investigations.

The heart contains a heterogeneous population of macrophages that could be divided into subsets having divergent origins, self-renewal capacities, and functional properties on the basis of TIMD4, CCR2, and MHC-II expression [6, 17, 46]. Our study is the first to assess the effects of age and MR deficiency on cardiac TIMD4^+^ and TIMD4^–^ macrophage populations. Consistent with previous studies in young mice [16, 17], the majority of macrophages was TIMD4^+^MHC-II^neg/low^. This declining macrophage population was progressively replaced by TIMD4^+^ and TIMD4^–^ macrophages expressing intermediate to high levels of MHC-II. Molawi et al., [46] using genetic fate mapping, parabiotic mice, and bone marrow chimeras, established that almost all embryo-derived macrophages at birth were MHC-II^neg/low^ and losing their capacity to self-renew with age are progressively replaced by MHC-II^pos^ macrophages deriving from circulating monocytes produced in the bone marrow. However, 2- to 9-month-old mice were used in the study of Molawi et al., [46], thus relatively young animals. Subsequent works using fate mapping, parabiosis, and single-cell RNA sequencing technologies demonstrated that cardiac tissue-resident TIMD4^+^MHC-II^neg/low^ macrophages are long-lived cells and were maintained through self-renewal and are not replaced by blood monocytes in adult mice (~ 20 weeks old) even after ischemic injury [16, 37]. However, microenvironmental signals can induce changes in macrophage phenotype independently of their origin. [30, 35, 37]. A recent study investigating tissue macrophage heterogeneity in mice up to 1 year of age demonstrated that all resident macrophage subsets upregulate MHC-II over time in a tissue-specific pattern [17]. Phenotypic changes of cardiac macrophages with aging, therefore, appears to reflect the gradual replacement of embryo-derived macrophages by infiltrating monocyte-derived macrophages as well as macrophage differentiation/polarization in response to changing local microenvironment.

Alterations in the number and relative proportion of cardiac macrophage subsets may have profound effects on the induction and maintenance of inflammaging. By integrating FACS analysis, cell sorting, and transwell co-culture experiments, we found that TIMD4^–^ macrophages were the most abundant macrophage population in the aged heart and responsible for the enhanced production of inflammatory cytokines. Moreover, we showed that the pro-fibrotic crosstalk between TIMD4^–^ macrophages and fibroblasts implies the macrophage MR and the release of mitochondrial superoxide anions. Specifically, our results indicate that aged TIMD4^–^ macrophages are mediators of the inflammatory and fibrotic responses. In a feed-forward loop, aged TIMD4^–^ macrophages stimulate fibroblasts to produce high levels of CCL2, IL-6, and collagen and fibroblasts trigger TIMD4^–^ macrophages to secrete CCL2 and IL-1ß. Notably, deficiency of the MR in aged TIMD4^–^ macrophages and the treatment with the mitochondria-targeted antioxidant MitoTEMPO similarly prevented the increase of superoxide anions generation as well as IL-6, CCL2, and IL-1ß secretion. Furthermore, immunofluorescence revealed that in the aged heart, TIMD4^–^ macrophages were mainly restricted to areas of fibrosis, which also contained a large amount of fibroblasts and showing strong immunoreactivity for collagen type I. Macrophage MR deletion resulted in a reduced accumulation of macrophages lacking TIMD4 along with significantly lower collagen type I expression in areas of fibrosis. These findings suggest the emergence of pro-fibrotic niches in the aging heart where dynamic interactions involving the macrophage MR take place between TIMD4^−^ macrophages and fibroblasts. In a very recent report (posted on Research Square), Lavine et al. [36] by ligand-receptor analysis and spatial transcriptomics predicted that interactions between CCR2^+^ macrophages and fibroblasts through the IL-1ß signaling drove the emergence of pro-fibrotic fibroblasts within spatially defined niches.

MCP-1/CCL2 is a major chemotactic cytokine that regulates the recruitment of mononuclear cells and plays an important role in stimulating monocyte activation and interstitial fibrosis [5, 65]. A relationship between increased CCL2 levels and enhanced macrophage recruitment in the senescent heart has been demonstrated [43]. In response to injury, upregulation of CCL2 mediates the recruitment of CCR2^+^ monocytes in the heart. CCL2-dependent monocyte recruitment has been shown to contribute to cardiac macrophage expansion in diastolic dysfunction induced by combining salty drinking water, unilateral nephrectomy, and chronic exposure to aldosterone [32]. Here, immunofluorescence showed that CCL2 was increased and localized to the fibrotic areas in aged hearts. Importantly, the reduced accumulation of TIMD4^–^ macrophages in old MR^LysMCre^ hearts was associated with a weak expression of CCL2 in PDGFRα-positive areas. We believe that blunted CCL2 secretion by TIMD4^–^ macrophages and fibroblasts in aged heart of MR^LysMCre^ mice likely lead to reduced expansion of the TIMD4^–^ macrophage population. Recently, Liu et al. [41] reported that high-fat diet-induced diastolic dysfunction was accompanied by increased cardiac CCL2 and an expansion in the TIMD4^−^CCR2^+^ macrophage population. In contrast to the restorative functions of embryo-derived resident TIMD4^+^ macrophages, several studies indicate that recruited monocyte-derived macrophages promote inflammation and worsen fibrosis [12, 31, 37]. Recently, in a pig model of reperfused acute myocardial infarction, fluorine-19 magnetic resonance imaging identified inflammatory monocytes/macrophages infiltration as independent determinant of cardiac contractile function and remodeling [12]. In different injury models, CCR2-deficient mice exhibit significantly reduced inflammation and fibrosis [32, 37]. Using single-cell transcriptomics analysis and fate mapping, Chakarov et al. [13] identified two populations of interstitial macrophages (LYVE1^hi^MHC-II^lo^ expressing TIMD4 and LYVE1^lo^MHC-II^hi^ lacking TIMD4) that are equally replenished by CCR2-dependent monocytes in adulthood and play a critical role in inflammation and fibrosis [13]. Of interest, the cardiac interstitial LYVE1^lo^MHC-II^hi^ macrophages highly express pro-inflammatory factors such as Il-1ß [13]. Recently, in an adoptive transfer model of Tet2-mediated clonal hematopoiesis, aged mice displaying a significant expansion of the monocyte-derived CCR2^+^MHC-II^hi^ macrophage population in the heart showed an accelerated decline in cardiac contractility along with increased fibrosis [64]. Overall, deletion of the macrophage MR may have positively affected the inflammatory and fibrotic responses associated with aging through an upstream mechanism involving lower expansion and pro-inflammatory activation of the TIMD4^–^ macrophage population, probably linked to a reduction in CCL2/CCR2-dependent macrophage recruitment.

Multiple studies demonstrated the pivotal role of macrophage MR signaling in heart fibrosis [5, 8, 11, 57] and that MR deletion in myeloid cells [7, 22] recapitulates the protective effects of MR antagonism on extracellular matrix turnover after injury in the heart and kidney. So far, pharmacological MR inhibition is well-consolidated therapeutic intervention for limiting fibrosis advancement in patients with heart failure and for preventing vascular and renal fibrosis [2, 9, 29, 47]. Our data suggest that targeting the macrophage MR and the (TIMD4^–^) macrophage–fibroblast crosstalk by MR antagonists might prevent inflammation and the expansion of fibrotic areas in the aged heart and thereby attenuate the progression of cardiovascular aging and associated morbidity. Dysregulation of MR signaling has been linked with vascular aging as well as hypertension, obesity, and diabetes in the elderly [29]. MR blockade with spironolactone exerts cardiovascular protective effects including reduction in fibrosis and inflammation in older people at increased risk of developing heart failure, as recently delineated in the HOMAGE (Heart OMics in AGEing) randomized trial [14, 20, 49]. Clinical data also suggest greater benefit of MR antagonism in obese female HFpEF (heart failure with preserved ejection fraction) patients compared to males [44]. Recent evidence revealed a central role of smooth muscle cell MR in obesity-associated coronary and cardiac dysfunction [18]. In obese female mice, MR deficiency in smooth muscle cells prevented cardiac inflammation and diastolic dysfunction independent of changes in aortic stiffening, adipose inflammation, metabolic dysregulation, and kidney injury [18]. Of note, in our study, transcriptome profiling of cardiac macrophages showed that protection against macrophage inflammation by MR deletion was shared between the sexes but more marked in MR^LysMCre^ female mice.

Some limitations and future directions should be considered**.** Heterozygous LysM^Cre^ mice express Cre in myeloid cells due to targeted insertion of the Cre cDNA into their endogenous Lyz2 (lysozyme M) locus. This results in Lyz2 deficiency in macrophages from MR^LysMCre^ mice. In line with several previous studies [7, 11, 22, 57], MR^flox^ mice were used as control animals. We cannot rule out off-target effects of partial Lyz2 loss and Cre transgene, and whether this have had an impact on the macrophage phenotype and led to a misinterpretation of results from MR^LysMCre^ mice. Lyz2 deficiency promoted more severe infection-induced inflammation [26]. Cre expression has been shown to induce DNA damage, apoptosis, and activation of pro-fibrotic genes, while chronic myocardial Cre expression leads to cardiotoxicity, fibrosis, and adverse remodeling [10]. Of interest, a recent study showed that deletion of Lyz2 in LysM^Cre^ recombinase homozygous mice is non-contributory in sterile acute lung injury [27]. However, further research is needed to assess whether LysM^Cre^ mice develop more severe macrophage inflammaging and whether reduced Lyz2 and prolonged Cre expression may have in part blunted the protective effects of MR deletion in macrophages during aging. Moreover, given that all systemic myeloid cells are affected by the LysM-Cre promoter, we cannot exclude that MR deficiency in macrophages in other tissues and reduced systemic inflammaging may have influenced the cardiac phenotype. However, our transcriptome profiling data and transwell experiments with cells sorted from young/old hearts support that MR activation promotes macrophage inflammaging and the inflammatory macrophage–fibroblast crosstalk in the heart. The development of novel approaches for selective targeting of macrophages in the myocardium is needed to better define the specific role of the macrophage MR in the aging heart. Furthermore, future studies are required to determine whether the cardioprotective effects of macrophage MR deficiency may be secondary to regulation of metabolic remodeling or arterial stiffness associated with aging.

In conclusion, our findings uncovering the pro-inflammatory and fibrogenic role of the macrophage MR in the context of cardiac aging could have implications for the prevention and treatment of inflammaging and age-related fibrotic disorders in the aging population.

### Supplementary Information

Below is the link to the electronic supplementary material.

Supplementary file1 (TIF 4432 KB)

Supplementary file2 (TIF 29462 KB)

Supplementary file3 (TIF 27026 KB)

Supplementary file4 (TIF 34594 KB)

Supplementary file5 (TIF 19054 KB)

Supplementary file6 (TIF 6011 KB)

Supplementary file7 (TIF 32612 KB)

Supplementary file8 (TIF 32944 KB) **Fig. S8**

Supplementary file9 (TIF 3019 KB)

Supplementary file10 (DOCX 17 KB)

Supplementary file11 (XLSX 25 KB)

## Data Availability

The data that support the findings of this study are available from the corresponding authors upon reasonable request.
