# Supplementary figures and images for: Mineralocorticoid receptor promotes cardiac macrophage inflammaging
Source: Basic Res Cardiol. 2024 Feb 8;119(2):243–60. doi: 10.1007/s00395-024-01032-6 (PMC11008080; doi:10.1007/s00395-024-01032-6)

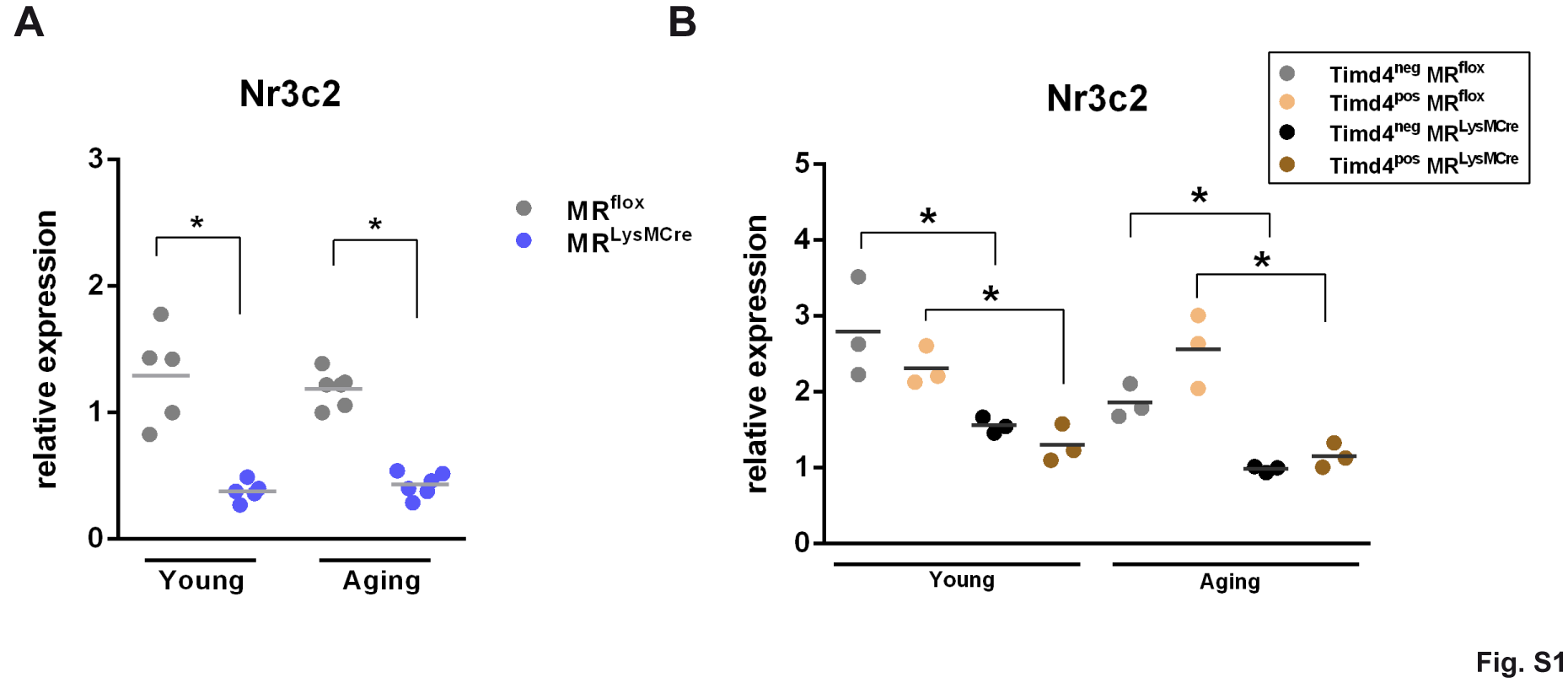

Supplement: Supplementary file 1 — Supplementary file1 (TIF 4432 KB) [file 395_2024_1032_MOESM1_ESM.tif]

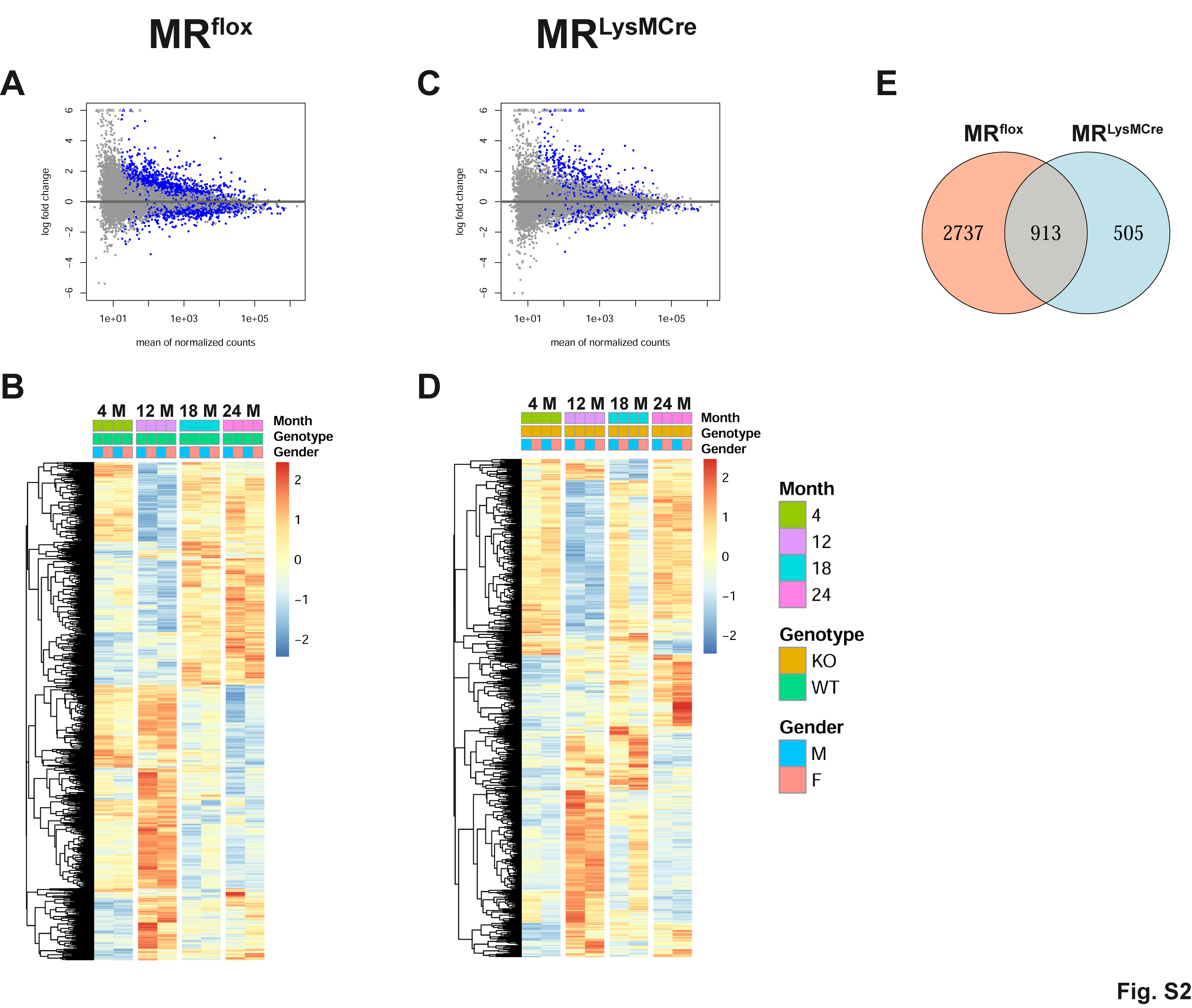

Supplement: Supplementary file 2 — Supplementary file2 (TIF 29462 KB) [file 395_2024_1032_MOESM2_ESM.tif]

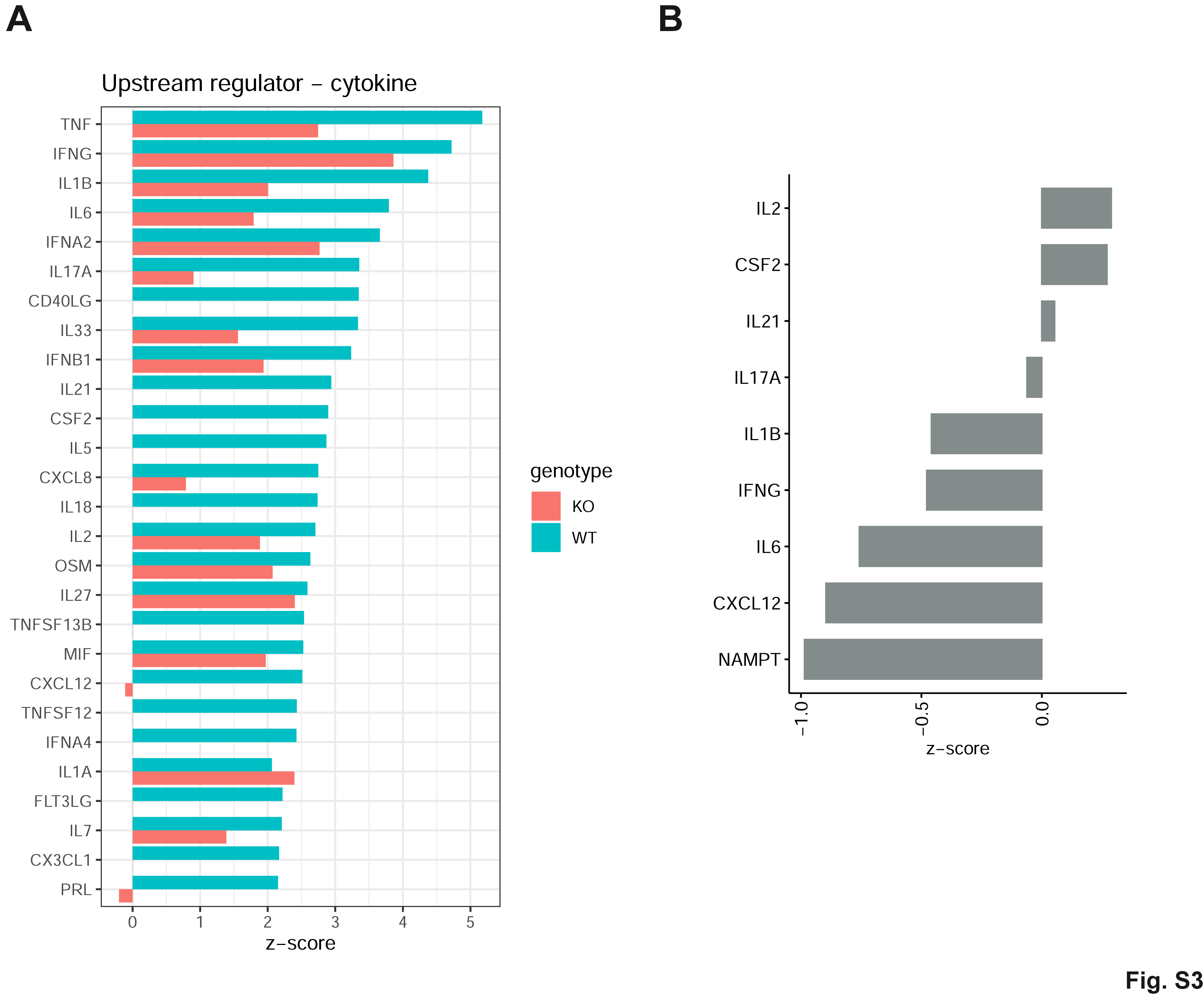

Supplement: Supplementary file 3 — Supplementary file3 (TIF 27026 KB) [file 395_2024_1032_MOESM3_ESM.tif]

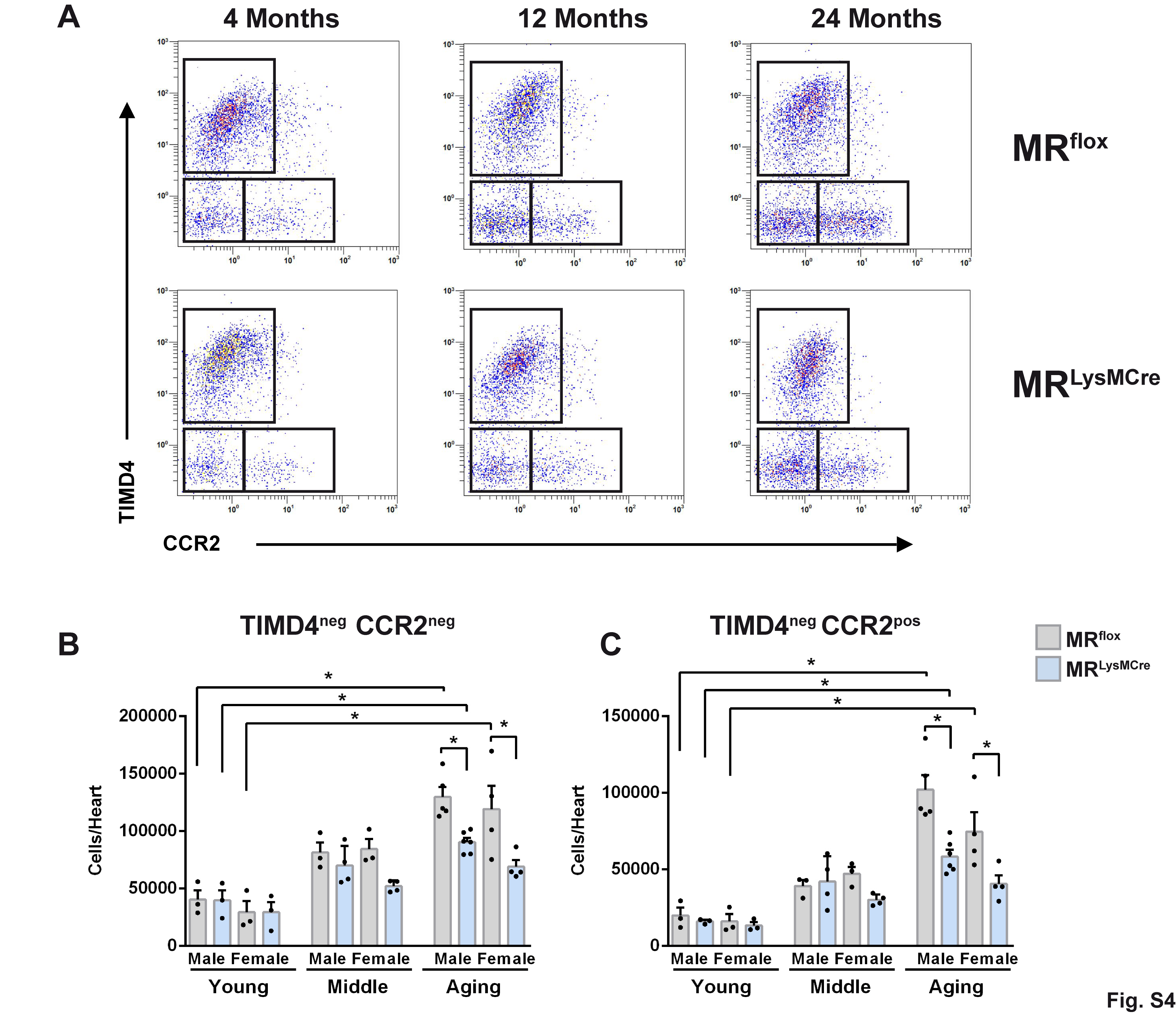

Supplement: Supplementary file 4 — Supplementary file4 (TIF 34594 KB) [file 395_2024_1032_MOESM4_ESM.tif]

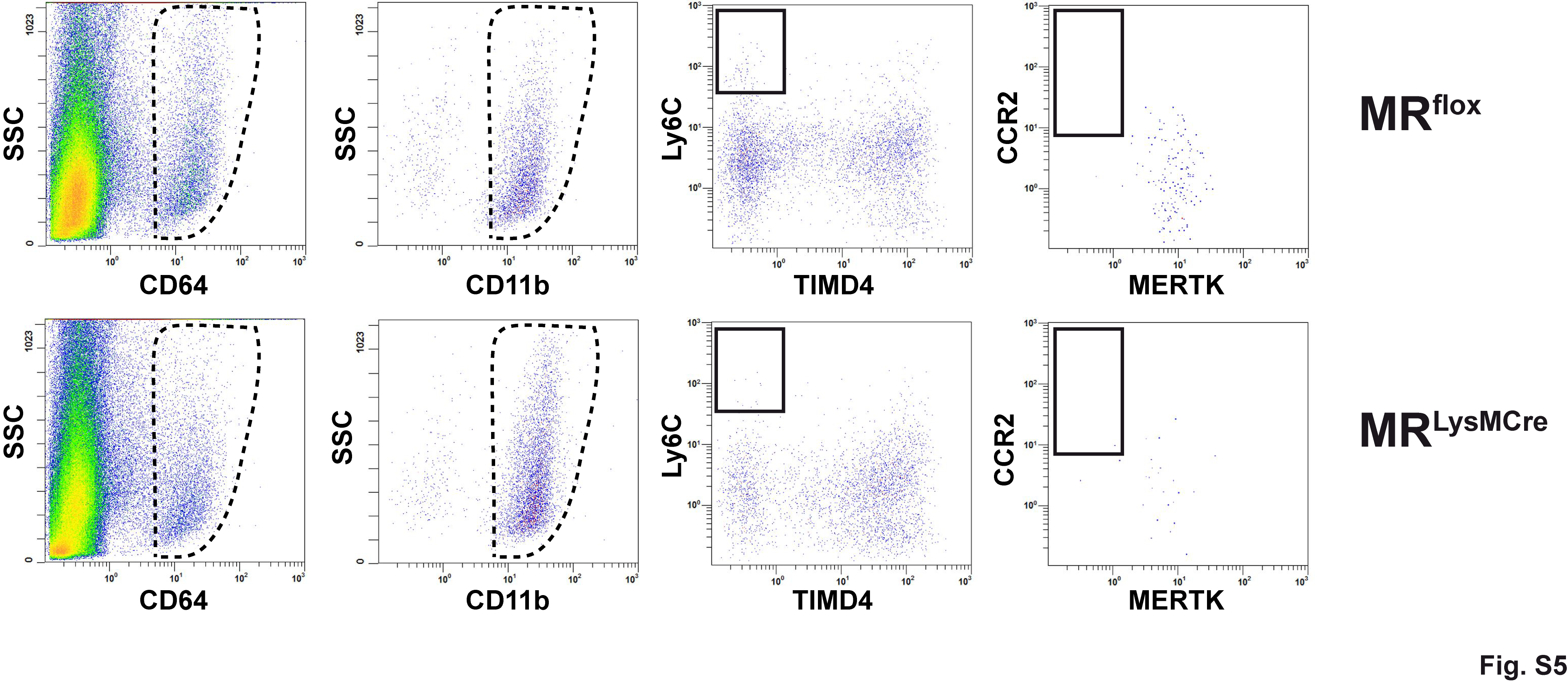

Supplement: Supplementary file 5 — Supplementary file5 (TIF 19054 KB) [file 395_2024_1032_MOESM5_ESM.tif]

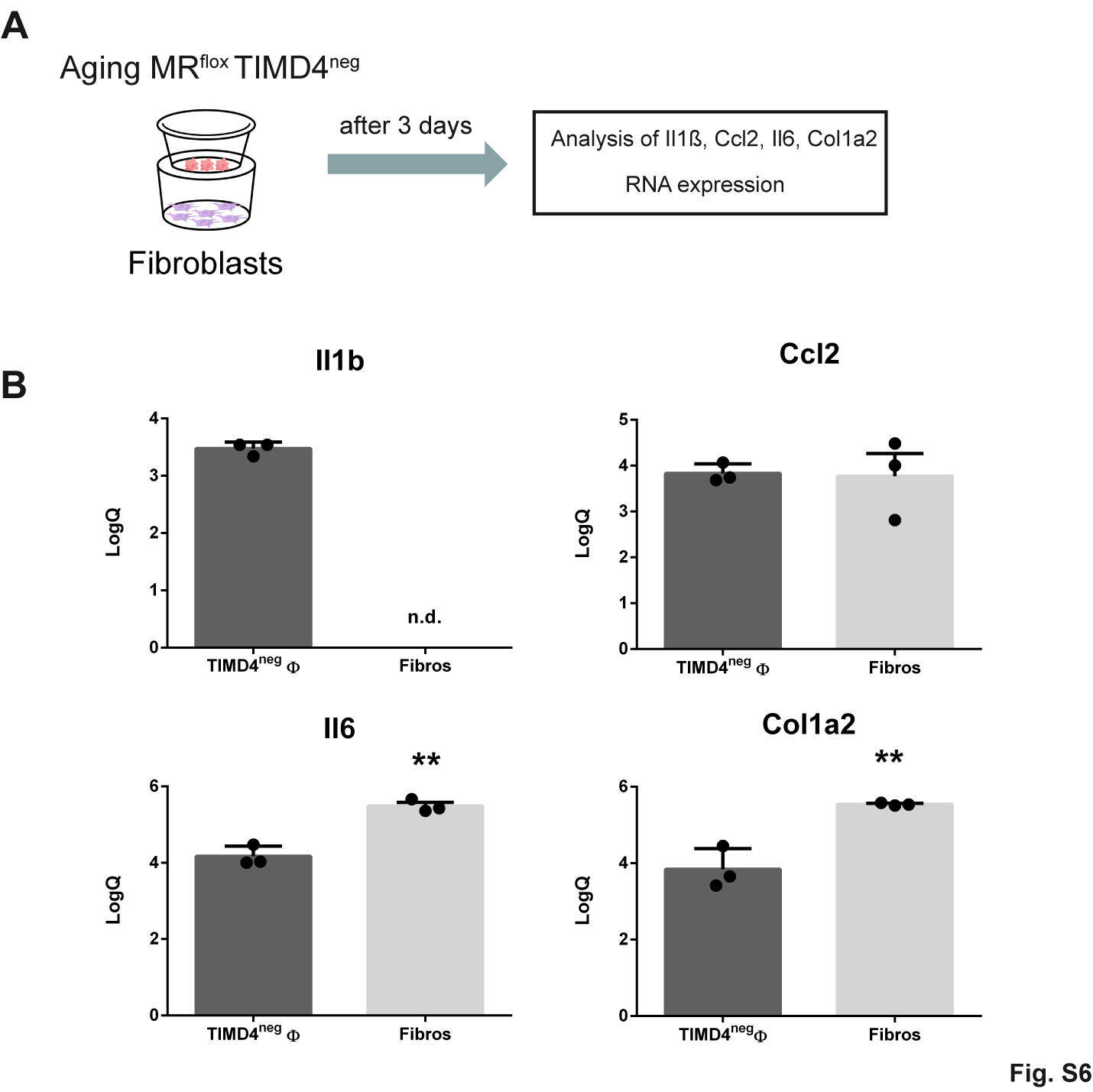

Supplement: Supplementary file 6 — Supplementary file6 (TIF 6011 KB) [file 395_2024_1032_MOESM6_ESM.tif]

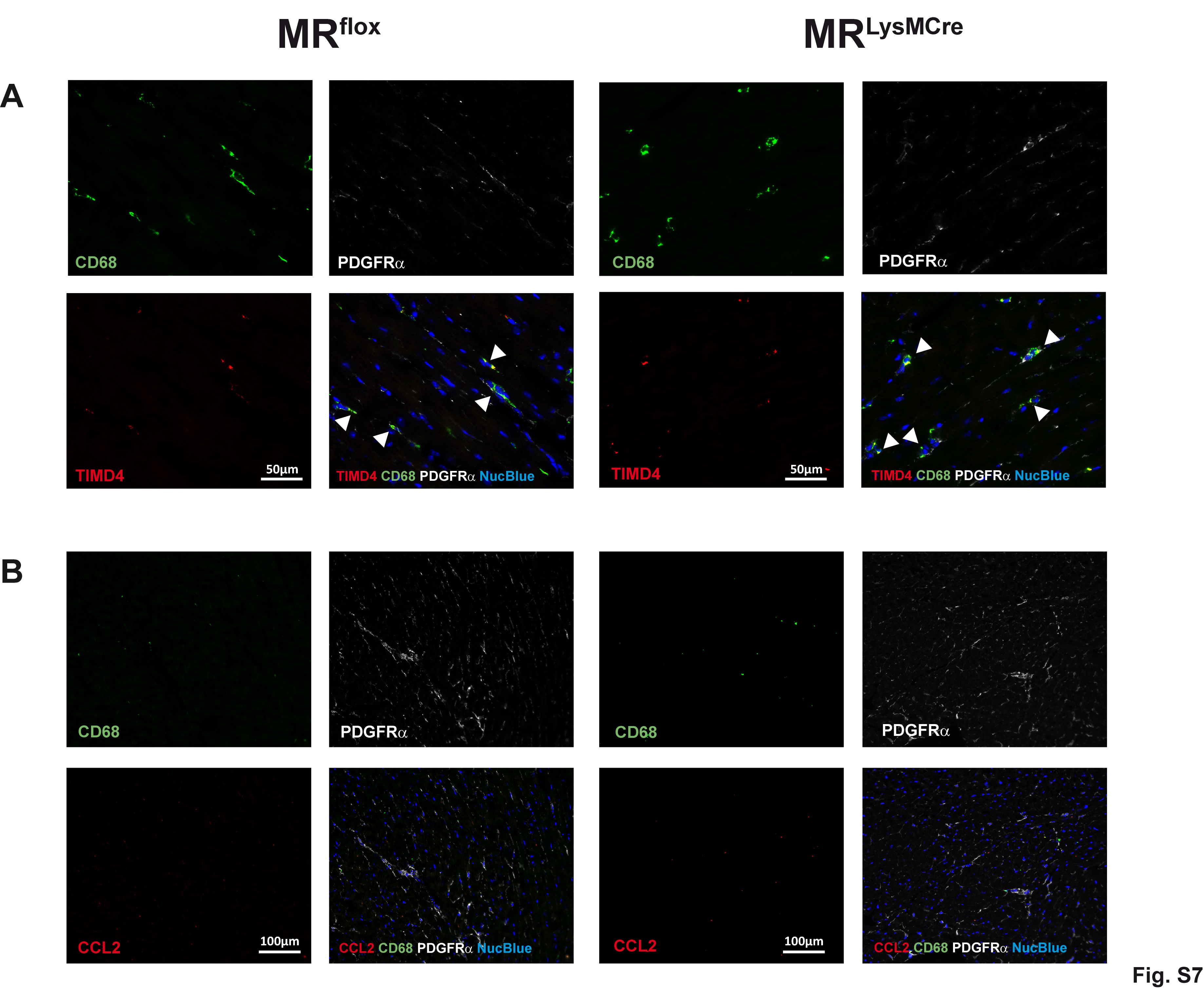

Supplement: Supplementary file 7 — Supplementary file7 (TIF 32612 KB) [file 395_2024_1032_MOESM7_ESM.tif]

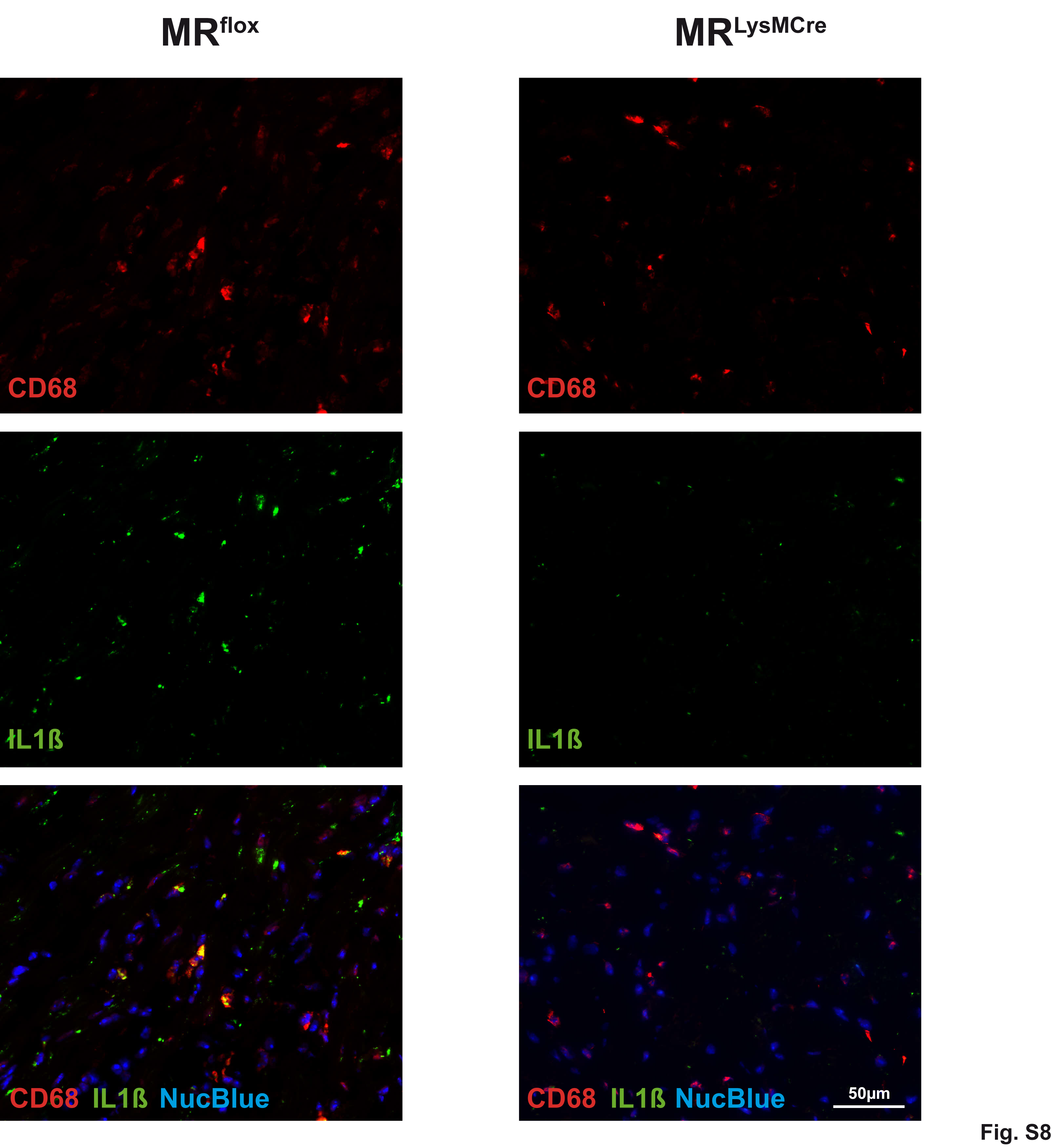

Supplement: Supplementary file 8 — Supplementary file8 (TIF 32944 KB) Fig. S8 [file 395_2024_1032_MOESM8_ESM.tif]

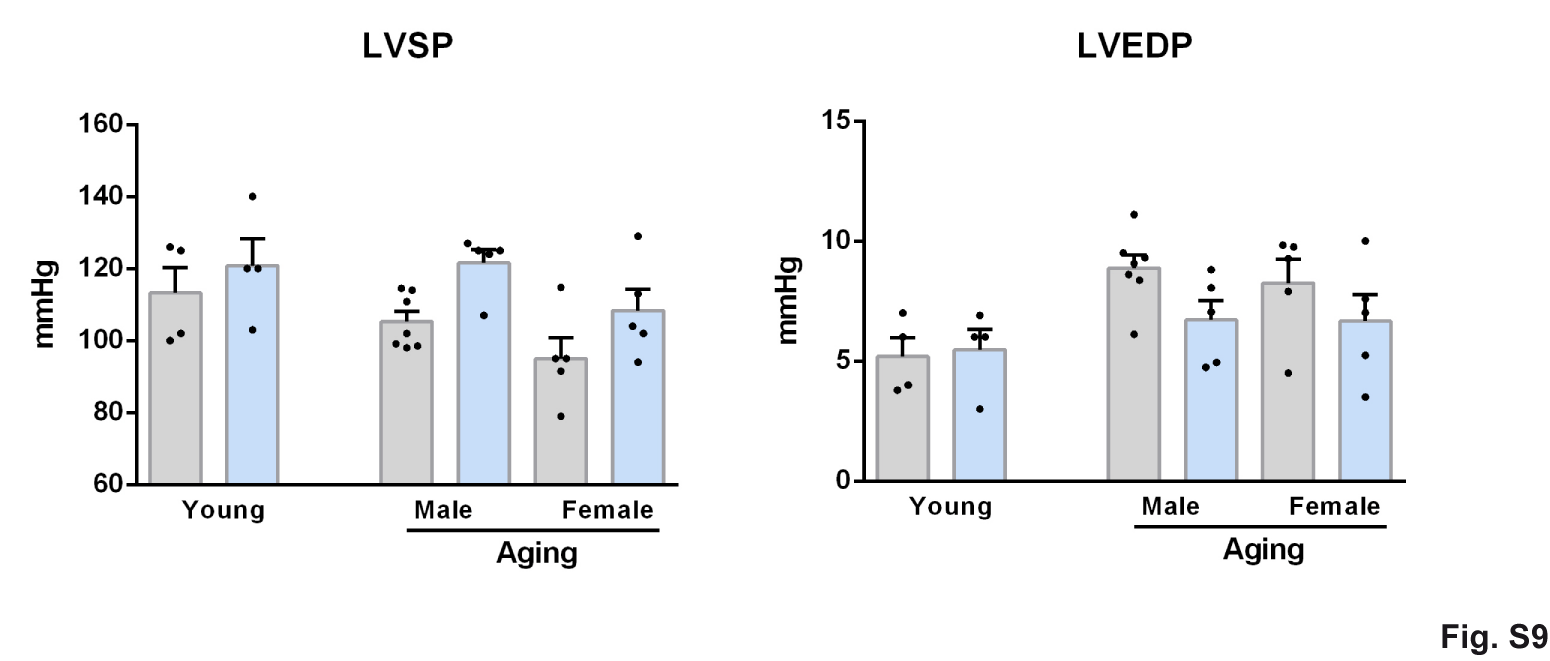

Supplement: Supplementary file 9 — Supplementary file9 (TIF 3019 KB) [file 395_2024_1032_MOESM9_ESM.tif]
