# Supplementary material for: Mineralocorticoid receptor promotes cardiac macrophage inflammaging
Source: Basic Res Cardiol. 2024 Feb 8;119(2):243–60. doi: 10.1007/s00395-024-01032-6 (PMC11008080; doi:10.1007/s00395-024-01032-6)
Supplement: Supplementary file 10 — Supplementary file10 (DOCX 17 KB) [file 395_2024_1032_MOESM10_ESM.docx]

**Legends to supplementary figures**

**Fig. S1**

Relative expression of the mineralocorticoid receptor in **A** CD45^+^/CD11b^+^/CD64^+^/MERTK^+^ macrophages and **B** CD45^+^/CD11b^+^/CD64^+^/MERTK^+^ macrophages lacking and expressing TIMD4 isolated by flow cytometry from young/old MR^flox^ and MR^LysMCre^ hearts. Mean±SEM, n = 3-6 per group; **p* < 0.05

**Fig. S2**

Time course analysis of RNA-Seq data performed with Likelihood ratio test (LRT). **A**, **C** MA-plots of gene expression over time in macrophages FACS-sorted from hearts of male/female young (4 months-old), middle-aged (12 months-old) and old (18 and 24 months-old) MR^flox^ and MR^LysMCre^ mice. Significantly differentially expressed genes (DEGs) with adjusted p-values < 0.1 generated by LRT are indicated in blue. **B**, **D** Heatmap showing *z* score normalized expression values of all significantly DEGs over time in macrophages from MR^flox^ and MR^LysMCre^ mice respectively. **E** Venn diagram of significantly DEGs (*p* < 0.1) of contrasts MR^flox^ and MR^LysMCre^  vs. corresponding baselines

**Fig. S3**

**A** Activated upstream regulators that may be involved in the regulation of the transcriptional profile of aged fibroblasts versus young fibroblasts from MR^flox^ and MR^LysMCre^ hearts. Cytokines such as IL6 and IL1b were predicted to be potentially upstream regulators of the transcriptional profile of aged cardiac fibroblasts. **B** Upstream regulators predicted to be inhibited in MR^LysMCre^ versus MR^flox^ aged cardiac fibroblasts. IPA was used to analyze RNA-seq data. Upstream regulators are ranked by *p* value of overlap. A negative *z* score indicates predicted inhibition, whereas a positive *z* score suggests predicted activation

**Fig. S4**

Macrophage MR deficiency reduced the expansion of the TIMD4^–^CCR2^–^ and TIMD4^–^CCR2^+^ macrophage subsets in the healthy aging heart. **A** Flow cytometric analysis of macrophages from hearts of young, middle-aged and old MR^flox^ and MR^LysMCre^ mice. Cardiac macrophages were identified as positive for CD45, CD11b, CD64 and stratified by CCR2 and TIMD4. FACS-based quantification of **B** cardiac TIMD4^–^CCR2^–^ and **C** TIMD4^–^CCR2^+^ macrophages. Mean±SEM, n = 3-6 per group; **p* < 0.05

**Fig. S5**

Flow cytometric analysis showing that monocytes were nearly absent in the aged hearts of MR^flox^ and MR^LysMCre^ mice. Heart monocytes were identified as CD64^+^, CD11b^+^, TIMD4^–^, CCR2^+^ cells expressing high levels of Ly6C and lacking MerTK expression

**Fig. S6**

**A** Experimental outline. Cardiac TIMD4^–^ macrophages and fibroblasts were isolated from hearts of old MR^flox^ mice and co-cultured for 3 days**. B** Il1b, Ccl2, Il6 and Col1α2 mRNA expression analyzed by absolute quantitative PCR. Mean±SEM, n = 3 per group; ***p* < 0.01

**Fig. S7**

Immunofluorescence micrographs of heart sections from young MR^flox^ and MR^LysMCre^ mice showing **A** macrophages (CD68^+^ cells), fibroblasts (PDGFRα^+^ cells) and TIMD4 immunoreactivity. Arrows indicate CD68^+^ and TIMD4^+^ cells. **B** Immunoreactivity for CCL2 in CD68 and PDGFRα-positive areas. The hearts from young MR^flox^ and MR^LysMCre^ mice displayed a weak expression of CCL2 in PDGFRα-positive areas. Nuclei were stained with NucBlue™

**Fig. S8**

Immunofluorescence micrographs of heart sections from old MR^flox^ and MR^LysMCre^ mice showing macrophages (CD68 positive cells) and IL-1ß immunoreactivity. Nuclei were stained with NucBlue™

**Fig. S9**

MR deficiency in myeloid cells has no impact on left ventricular systolic or diastolic pressure measured in vivo with a conductance catheter in young and old MR^flox^ and MR^LysMCre^ mice. Aged MR^flox^ and MR^LysMCre^ mice were on average 22 (±0.7) months old. Mean±SEM, n = 3-7 per group; **p* < 0.05
